# Candidate Key Proteins in Tinnitus: A Bioinformatic Study of Synaptic Transmission in Spiral Ganglion Neurons

**DOI:** 10.1007/s10571-023-01405-w

**Published:** 2023-09-22

**Authors:** Johann Gross, Marlies Knipper, Birgit Mazurek

**Affiliations:** 1 Tinnitus Center, Charité-Universitätsmedizin Berlin, Berlin, Germany; 2 Department of Otolaryngology, Head and Neck Surgery, Tübingen Hearing Research Center (THRC), Molecular Physiology of Hearing, University of Tübingen, Tübingen, Germany; 3 Leibniz Society of Science Berlin, Berlin, Germany

**Keywords:** Perception of sound, Acoustic stimulation, Key proteins, Spiral ganglion, Synaptic transmission, Tinnitus

## Abstract

To study key proteins associated with changes in synaptic transmission in the spiral ganglion in tinnitus, we build three gene lists from the GeneCard database: 1. Perception of sound (PoS), 2. Acoustic stimulation (AcouStim), and 3. Tinnitus (Tin). Enrichment analysis by the DAVID database resulted in similar Gene Ontology (GO) terms for cellular components in all gene lists, reflecting synaptic structures known to be involved in auditory processing. The STRING protein–protein interaction (PPI) network and the Cytoscape data analyzer were used to identify the top two high-degree proteins (HDPs) and their high-score interaction proteins (HSIPs) identified by the combined score (CS) of the corresponding edges. The top two protein pairs (key proteins) for the PoS are BDNF-GDNF and OTOF-CACNA1D and for the AcouStim process BDNF-NTRK2 and TH-CALB1. The Tin process showed BDNF and NGF as HDPs, with high-score interactions with NTRK1 and NGFR at a comparable level. Compared to the PoS and AcouStim process, the number of HSIPs of key proteins (CS > 90. percentile) increases strongly in Tin. In the PoS and AcouStim networks, BDNF receptor signaling is the dominant pathway, and in the Tin network, the NGF-signaling pathway is of similar importance. Key proteins and their HSIPs are good indicators of biological processes and of signaling pathways characteristic for the normal hearing on the one hand and tinnitus on the other.

**Graphical Abstract:**

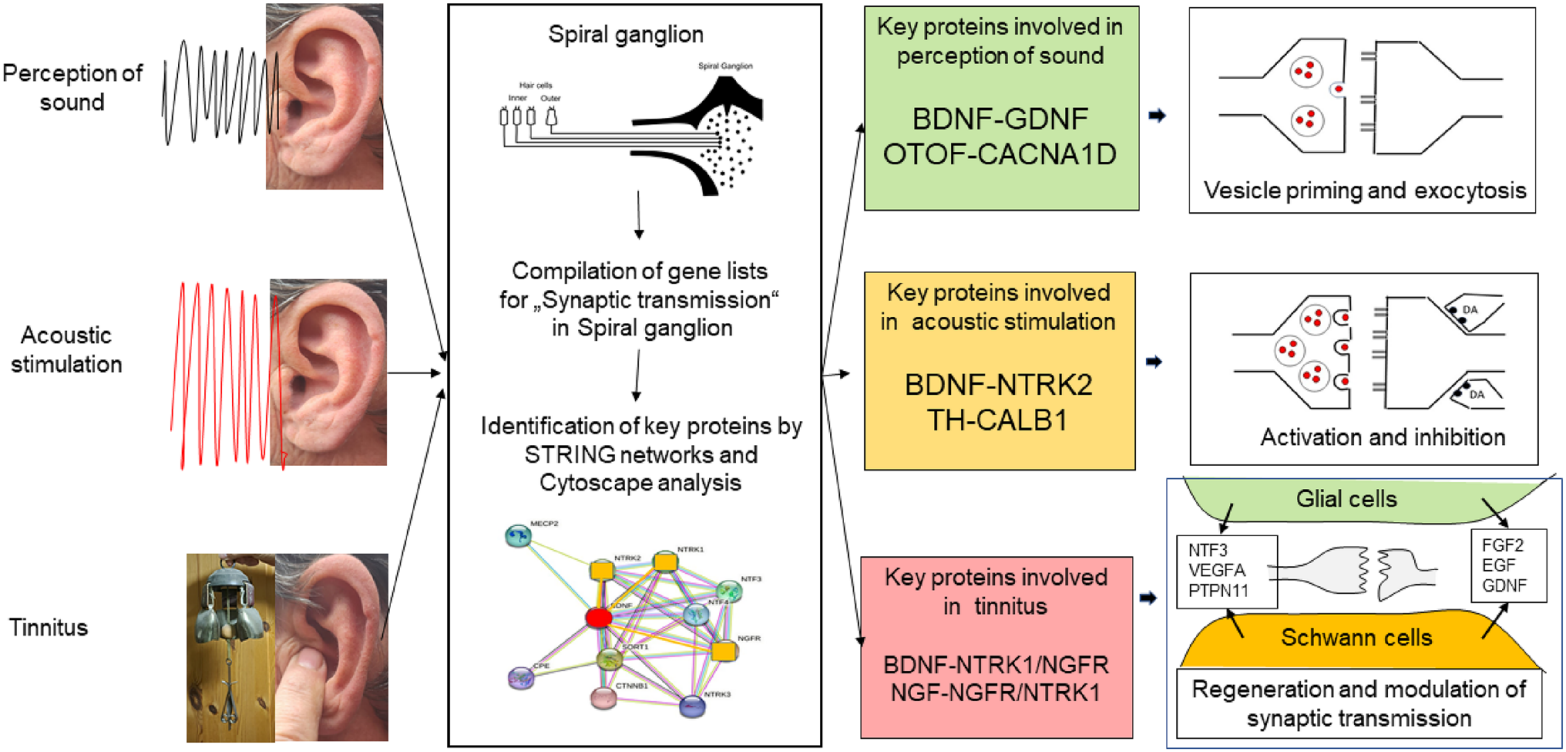

## Introduction

Tinnitus is the subjective perception of sounds when objectively no physical stimuli are present. The word derives from the Latin verb tinnire (to ring); this phenomenon affects about 15% of the population. Depending on the severity, tinnitus can be a very large psychological and disease burden on the afflicted individual and result in enormous health care costs (Baguley et al. 2013; Tziridis et al. 2022).

Common causes for tinnitus are hearing loss, noise damage, toxic substances, such as aminoglycoside antibiotics or cisplatin, infections, genetic reasons, aging processes, and neurological, cardiovascular, metabolic, and psychological diseases (Baguley et al. 2013; Zhang et al. 2021). The most common risk factor is hearing loss due to damage to hair cells in the cochlea, leading to adaptive changes in the activity of neurons along the neural auditory axis. The causes of tinnitus induce changes in activity-dependent synaptic plasticity of the neural network due to homeostatic regulation (Turrigiano 2012). As a consequence of permanent hearing loss or auditory fiber loss, the imbalance between excitation and inhibition leads to changes in neurotransmission and of neuronal regulation in the auditory cortex. This involves changes in the metabolic pathways, protein synthesis, and protein–protein interactions.

Different mechanisms underlying the development of tinnitus have been discussed (Eggermont and Roberts 2015; Sun et al. 2018; Knipper et al. 2020; Rupert and Shea 2022; Isler et al. 2022): (1) Tinnitus is the result of altered compensatory hyperexcitability in the central nervous system (CNS). It is assumed that in response to deafferentation of inner hair cells changed synapse formation between parvalbumin-positive interneurons and projecting neurons in the ascending auditory pathway are the basis for hyperexcitability. Parvalbumin-positive neurons belong to the class of GABA-ergic inhibitory neurons of the CNS with high nerve conduction velocity. (2) Tinnitus is the result of deficits in homeostatic increases in central neural gain due to a disturbance of neural synchrony. Neural synchrony is essential for auditory processing under difficult listening conditions. (3) A reduced firing rate due to defective hair cells leads to an increased spontaneous activity in the auditory centers of the CNS. This imbalance, when a reduced signal leads to an increased spontaneous response, is linked to deficits in central gain resulting in central noise, a feature that is interpreted in the context of a reduced tonic inhibition in the central neurons (Zeng 2020; Knipper et al. 2020). All three theories have in common that tinnitus causes structural and functional remodeling in the auditory nuclei of the CNS. These changes begin with damage to the inner hair cells and processing changes or degeneration of the spiral ganglion neurons (SGNs), which induces changes in central synaptic transmission and plasticity in all auditory centers. Here, particular activity-driven proteins as, e.g., brain-derived nerve growth factor (BDNF) have been suggested to display a fundamental role for altered excitability in the ascending peripheral auditory systems and central auditory signal processing in auditory centers during pathologies as tinnitus (Knipper et al. 2020, 2022).

Spiral ganglion neurons (SGN) are the first neurons that process acoustic signals from hair cells through action potentials. Neurons of the spiral ganglion (SG) are bipolar; their dendrite innervates a hair cell of the organ of Corti and their axon projects to the ventral and dorsal cochlear nuclei (Nayagam et al. 2011; Pavlinkova 2020). The neurons of the SG show a high diversity (Sun et al. 2018), including type I SGN and type II SGN, and diverse subtypes. The SGN are organized according to frequency, reflecting the tonotopic organization. Damage to hair cells leads to functional and structural changes in SG neural activity (Lefebvre et al. 2002; Zhang et al. 2021). With acoustic overload, hair cell damage is detectable within minutes to hours or days. By contrast, SGN degeneration occurs very slowly, over months and years (Hickman et al. 2021). The temporal differences in the degeneration time of inner hair cells on the one hand and SGN on the other indicate that the cell death of SGN is a secondary and long-lasting event. Most transmission of afferent signals from inner hair cells to postsynaptic neurons in the brainstem is carried out by type I SGNs. These cells are also the most sensitive to noise and to damaging processes due to aging or drugs (Resnik and Polley 2021). In humans, about 40% of these neurons show degeneration after the age of 50 (Wu et al. 2019).

Synaptic transmission is a fundamental process in normal hearing and tinnitus and describes communication between neurons based on synapses; it can be chemical or electrical. Chemical transmission occurs via the presynaptic release of neurotransmitters and their postsynaptic binding to receptors. Electrical synaptic transmission occurs via gap junctions and ion channels. These biological processes are made possible by the precisely regulated interaction of numerous proteins, described as the protein–protein interaction (PPI) network. So far, too little is known about which proteins play a key role in these processes. The identification of candidate key proteins and the analysis of molecular networks has become increasingly possible through the methods of bioinformatics and extensive freely accessible databases. The aim of the present study was to identify key proteins in SG, the first complex of neurons that process disturbances in hair cell signaling that can lead to tinnitus.

## Materials and Methods

To assess the differences in synaptic transmission in tinnitus and normal perception of sounds at the molecular level, we chose the following approach: 1. Three gene lists were compiled from the GeneCard database (GC; https://www.genecards.org/; Stelzer et al. 2016) for the following keywords: (a.) “perception of sound”; (b.) “Acoustic stimulation”; and (c.) Tinnitus; in addition the key words “synaptic transmission” AND “spiral ganglion” were used (13 June 2023). 2. The gene lists “Perception of sound” (PoS, Appendix 1), “Acoustic stimulation” (AcouStim, Appendix 2), and tinnitus (Tin, Appendix 3) were characterized by analyses of gene overlap using Venn diagrams (http://bioinformatics.psb.ugent.be/webtools/Venn/) and identification of Gene Ontology (GO) terms using the Database for Annotation, Visualization, and Integrated Discovery (DAVID; https://david.ncifcrf.gov/; Sherman et al. 2007). 3. The construction of PPI networks was performed using the STRING database (Search Tool for the Retrieval of Interacting Genes; https://string-db.org/; Szklarczyk et al. 2019). For both the DAVID and STRING database analyses, *Homo sapiens* was used as the species. 4. The Cytoscape data analyzer was used in the PPI network to identify the top two proteins (nodes) with the highest degree and the corresponding proteins characterized by high-score interactions (Shannon et al. 2003; Ashtiani et al. 2018; https://cytoscape.org/). Because of different biases within the lists of genes and proteins, the top two protein pairs were selected for analysis. As criteria for key proteins, the degree of nodes (HDPs-high-degree proteins), the Betweenness Centrality and the Closeness Centrality were used. As a guide to the corresponding high-score interaction proteins (HSIP), we used the Combined Score (CS) of the corresponding edges. This score includes, among others, the coexpression, the experimentally determined interaction, and the automated text mining. The top two HDPs and HSIPs are subsequently referred to as key proteins. We hypothesized that these protein pairs play an important role in the activity of pathways associated with the perception of sound or tinnitus. 5. The KEGG database was used to identify the molecular pathways in which key proteins and their HSIP act together (https://www.genome.jp/kegg/pathway.html). The following databases were used to define or briefly characterize proteins or genes: https://www.ncbi.nlm.nih.gov/genbank/; https://www.genenames.org/; https://www.uniprot.org/uniprotkb/; https://syngoportal.org; and https://thebiogrid.org/.

## Results

### Characterization of Gene Lists

The number of genes identified as relevant for the selected terms differed between the lists: 36 genes for PoS, 34 for AcouStim, and 61 for Tin (Appendices 1–3). There are apparently more genetic studies of the SG with regard to tinnitus as a disease than for PoS or AcouStim as physiological auditory functions. The numerical value of the score as an indicator of the relevance of the gene for the indicated processes is similar for all gene lists and ranged from 0.8 to 30.9.

**Fig. 1:**
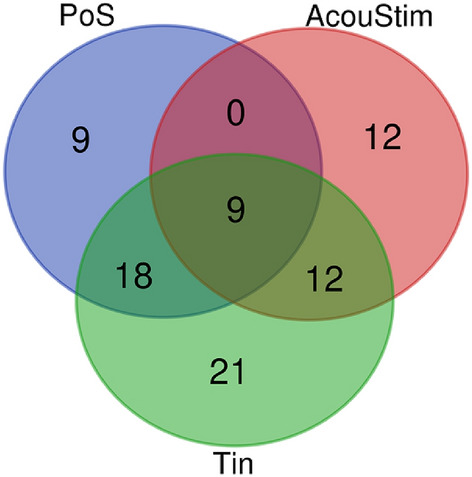
Venn diagram of three gene lists. Overall number of unique elements: 81. The overlapping genes see Table 1

To characterize the three gene lists PoS, AcouStim, and Tin, the analyses of overlap in the Venn diagram and enrichment of GO terms were used. The overall number of unique genes of all lists in the Venn diagram was nine (Fig. 1). The distribution of genes among the different groups of the Venn diagram is summarized in Table 1. The nine genes detectable in PoS, AcouStim, and Tin group included seven genes that can be assigned to the GO term “sensory perception of sound.” The number of genes unique to Tin is nearly twice as large as the number of genes detectable in PoS or AcouStim.

**Table 1:** Distribution of genes of the three gene lists among the different groups of the Venn diagram

AcouStim + PoS + Tin (9)	PoS + Tin (18)	AcouStim + Tin (12)	PoS (9)	AcouStim (12)	Tin (21)
BDNF	CA2	CALB2	CPA6	CERNA3	MIR210
CACNA1D	MBP	MAP2	MPDZ	NTRK2	GPHN
P2RX2	GDNF	NOS2	GRM8	NOS1	NTF3
RET	TBC1D24	MALAT1	CHAT	CYP19A1	PCAT1
OTOF	WHRN	EGF	ASIC2	CNTF	PANX1
TH	SLC17A8	NTS	MT-CO3	SOD2-OT1	HIF1A
CDH23	MAP1B	JUN	LMNB1	TMX2-CTNND1	NTRK1
SLC26A5	GRM7	NRG1	NOG	PVALB	FGF2
KCNQ4	MYO7A	SYP	DES	SMAD5-AS1	ID1
	KCNJ10	NR3C1		CALB1	CAMK2G
	PTPN11	CREB1		MAPK10	ANXA5
	SNAP25	GFAP		OPRM1	VEGFA
	SETD2				CACNA1G
	NRTN				NGFR
	MAP2K1				GFRA1
	BTD				USF1
	MAFB				NGF
	GJA1				NTRK3
					VIM
					NUDT6
					S100B

For the DAVID enrichment analysis, the top five GO terms “cellular components” (CC) and “biological processes” (BP) were selected (Table 2). The number of significant charts in the PoS gene set was 40, in the AcouStim gene set 63, and in the Tin gene set 109 (at a *p* value of < 0.01). To limit the analysis, only the top five GO terms with the highest significance will be mentioned and discussed below. The top five CC-GO terms of the PoS, AcouStim, and Tin gene lists reflect neural structures known for auditory processing, with only small differences between the gene lists. The top five BP-GO terms of the PoS gene list reflect terms that are characteristic for the normal hearing process. The top five BP-GO terms for the AcouStim gene list are indicative of the normal hearing process, but also of the activation of acoustic processes (e.g., response to lipopolysaccharide, response to hypoxia). The top five BP-GO terms for the Tin gene list are indicative of changes in regulation of gene expression and growth processes. In general, there is some overlap and missing specificity in the GO terms.

**Table 2:** Top five Cellular components (CC) and Biological Processes (BP) GO Terms in the spiral ganglion for PoS, AcouStim, and Tin

PoS (40 charts)	AcouStim (63 charts)	Tin (109 charts)
*CC (p* = *3.5E-4 to 1.5 E-3; fold enrichment 2–51)*	*CC p* = *2.9E-8 to 1.1 E-3; fold enrichment 17–60)*	*CC p* = *7.1E-7 to 1.1 E-3; fold enrichment 8–12)*
Axon (6, 16%)	Axon (9, 27%)	Axon (9, 20%)
Neuron cell body (6, 16%)	Terminal bouton (5, 15%)	Dendrite (7/15%)
Plasma membrane (20, 54%)	Dendrite (8, 24%)	Presynapse (5/11%)
Dendrite (6, 16%)	Neuron projection (6, 18%)	Gap junction (3/7%)
Stereocilium (3, 8%)	Stereocilium (3, 9%)	Neuron cell body (6/13%)
*BP (p* = *7.0E-17 to 2.0E-3; fold enrichment 16–83)*	*BP (p* = *1.8E-7 to 1.2 E-3); fold enrichment 7–27)*	*BP (p* = *1.6E-6 to 2.5E-5; fold enrichment 20–30)*
Sensory perception of sound (13, 35%)	Sensory perception of sound (7, 21%)	Positive regulation of gene expression (13/28%)
Auditory receptor cell stereocilial organization (3, 8%)	Response to hypoxia (5, 15%)	Sensory perception of sound (7/15%)
Peripheral nervous system development (3, 8%)	Cochlear development (3, 9%)	Bergmann glial cell differentiation (4/9%)
Neuron projection development (4, 11%)	Positive regulation of protein kinase B signaling (4, 12%)	Neuron projection development (6/13%)
Regulation of transmembrane ion transport (4, 11%)	Positive regulation of gene expression (6, 18%)	Positive regulation of ERK1 and ERK2 cascade (7/15%)

In brackets: Numbers of genes per GO terms (absolute and in %). Order of the GO terms according to the p values

### Key Proteins and Their High-Score Interaction Proteins of the Pos, AcouStim, and Tin Network

Network analysis is suited to identify influential candidate proteins using topological criteria of nodes and their interactions by edges. With the exception of Brain-Derived Neurotrophic Factor (BDNF), which is present in all analyzed terms, key proteins clearly differed between PoS, AcouStim, and Tin processes (Table 3). Key proteins for the PoS term were BDNF, Glial Cell-Derived Neurotrophic Factor (GDNF), Otoferlin (OTOF), and Calcium Voltage-Gated Channel Subunit Alpha1 D (CACNA1D), for the term AcouStim were BDNF, Neurotrophic Receptor Tyrosine Kinase 2 (NTRK2), Tyrosine Hydroxylase (TH), and Calbindin 1 (CALB1, and for Tin term BDNF, Neurotrophic Receptor Tyrosine Kinase 1 (NTRK1), Nerve Growth Factor (NGF), and Nerve Growth Factor Receptor (NGFR). It is important to note that in the tinnitus group there were also close associations of BDNF to NGFR and of NGF to NTRK1 (Table 3).

**Table 3:** Key proteins in the networks of spiral ganglion in the PoS, AcouStim, and Tin groups

HDP	Degree	Close	Betw	HSIP	Coexp	Exp	Text	CS
*PoS*								
BDNF	28	0.59	0.29	GDNF	0	0	950	950
OTOF	22	0.54	0.22	CACNA1D	62	0	903	905
*AcouStim*								
BDNF	38	0.74	0.14	NTRK2	65	762	995	999
TH	34	0.70	0.07	CALB1	62	0	810	814
*Tinnitus*								
BDNF	64	0.65	0.10	NTRK1^a^	62	76	990	999
NGF	62	0.64	0.08	NGFR^b^	0	792	990	999

*Close* closeness centrality, *Betw* betweenness centrality, *HDP* high-degree protein, *HSIP* high-score interaction protein, *Coexp* coexpression, *Exp* experimentally determined interaction, *Text* automated text mining, *CS *combined score

^a^HSIs with a CS = 999 also with NGFR

^b^HSIs with a CS = 999 also with NTRK1

### Networks of the PoS, AcouStim, and Tin Processes

**Fig. 2:**
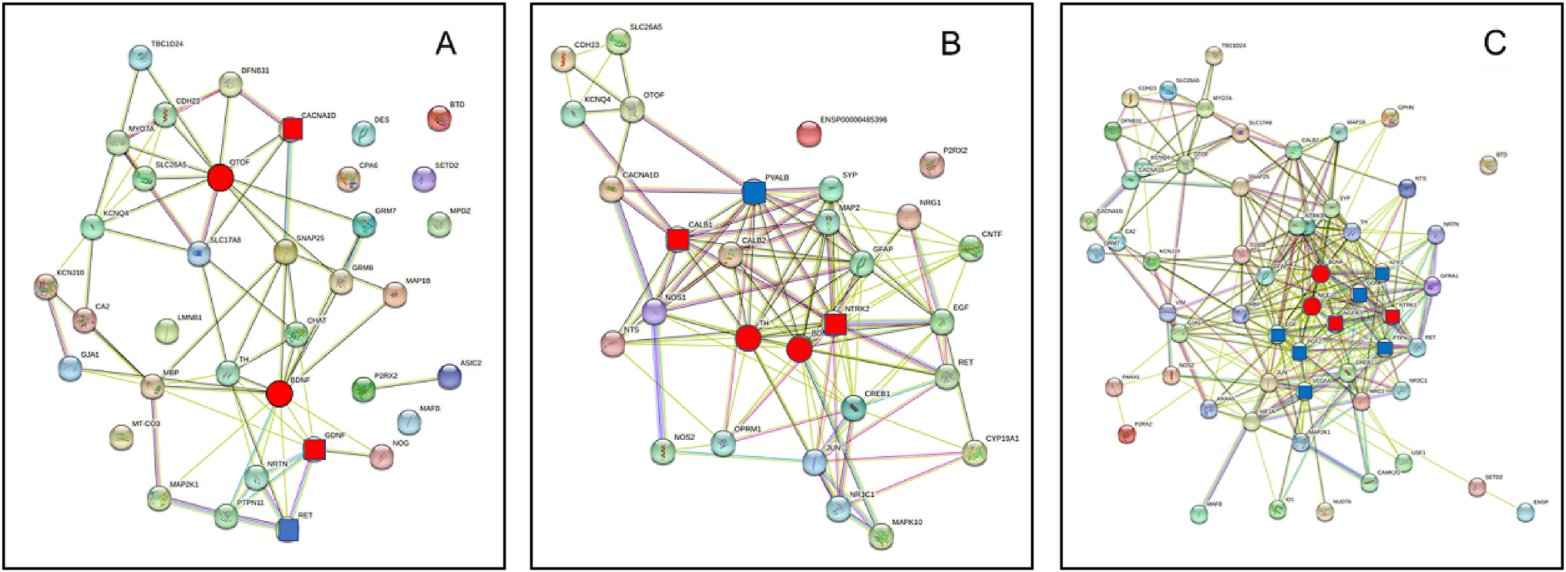
PPI networks of the PoS, AcouStim, and tinnitus processes in the SG. **A** PoS; **B** AcouStim; **C** Tinnitus. Number of nodes (PoS/AcouStim/Tin-28/ 27/57); number of edges-140/236/666). Top1 and top2 key proteins—red. Circles—HDPs, square—HSIPs. HSIPs according to Fig. 4 blue

Figure 2 illustrates the structure of the PoS, AcouStim, and Tin networks and the localization of the top two HDPs and HSIPs in the networks. The number of proteins for each network is smaller than the number of genes (Appendices 1–3); this is caused by the lack of interaction of the corresponding proteins in the PPI network or the presence of genes that encode transcripts. In the Tin list, this concerns the genes BDNF Antisense RNA (BDNF-AS), Biotinidase (BTD), Metastasis-Associated Lung Adenocarcinoma Transcript 1 (MALAT1), MicroRNA 210 (MIR210), and Prostate Cancer-Associated Transcript 1 (PCAT1). BDNF-AS, with a very high tinnitus score (30.9), exerts its effects via the regulatory mechanism of microRNA (Yuksel et al. 2023). In general, the top1 and top2 HDPs are close together in their networks.

For the quantitative characterization of the networks, we used topological parameters and the relative frequency distribution of the degrees of the nodes and of the combined score values of the edges. The topological parameters differ slightly (PoS/AcouStim/Tin): The average number of neighbors (5.0/8.7/11.7) and the network diameter (4/5/6) indicate that the average connectivity of the nodes in the Tin network is somewhat higher than in the PoS and AcouStim networks. The network heterogeneity (0.5/0.5/0.7) indicates some differences between PoS/AcouStim on one hand and Tin on the other hand. The characteristic path length values (2.2/1.9/2.2) and the clustering coefficient (0.6/0.7/0.6) tend to be different in the AcouStim network compared to the PoS and Tin networks.

**Fig. 3:**
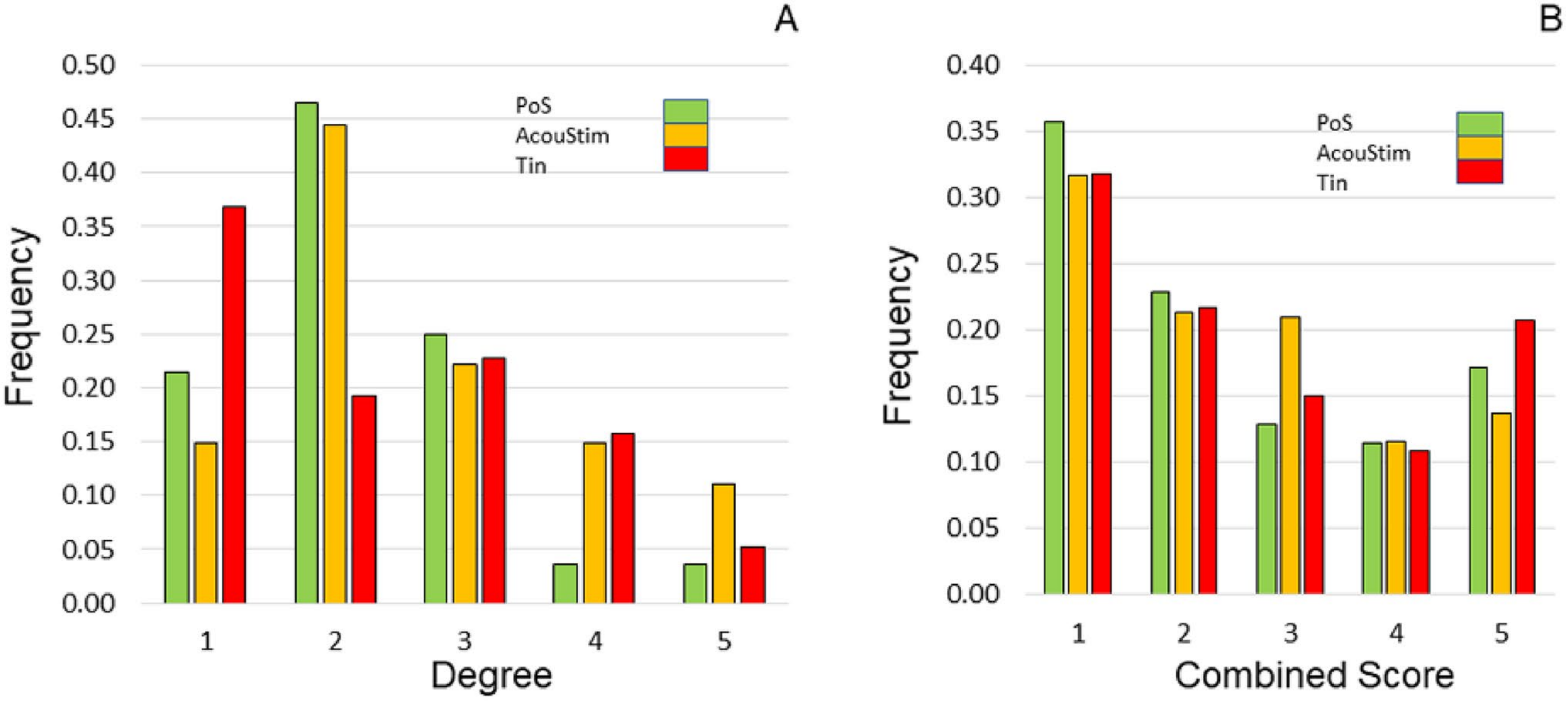
Relative frequency distribution of degrees of nodes (**A**) and of the CS of edges (**B**) of the PoS, AcouStim, and Tin protein–protein networks. Five classes of equal size were built. **A** Degree values were normalized to the highest degree (PoS/AcouStim/Tin = 28/27/57), and frequency values were normalized to the number of proteins in the network (27/26/54). **B** CS values are a function of the genes involved and were used as absolute values. Classes include CS values of 120; Class 5 includes CS values from 880 to 999. Frequency was normalized to the number of interactions in the network (140/236/666)

For characterizing the distribution of degree values of the nodes and the CS of the edges in the three networks, we calculated the relative frequency of degree and CS values (Fig. 3a, b). The abundance of CS values shows a valley at class 4 values (760–880); that is, the abundance of protein pairs with class five values is significantly higher. In the PoS and Tin groups, the frequency of protein pairs with high CS values is higher than in the AcouStim group.

**Fig. 4:**
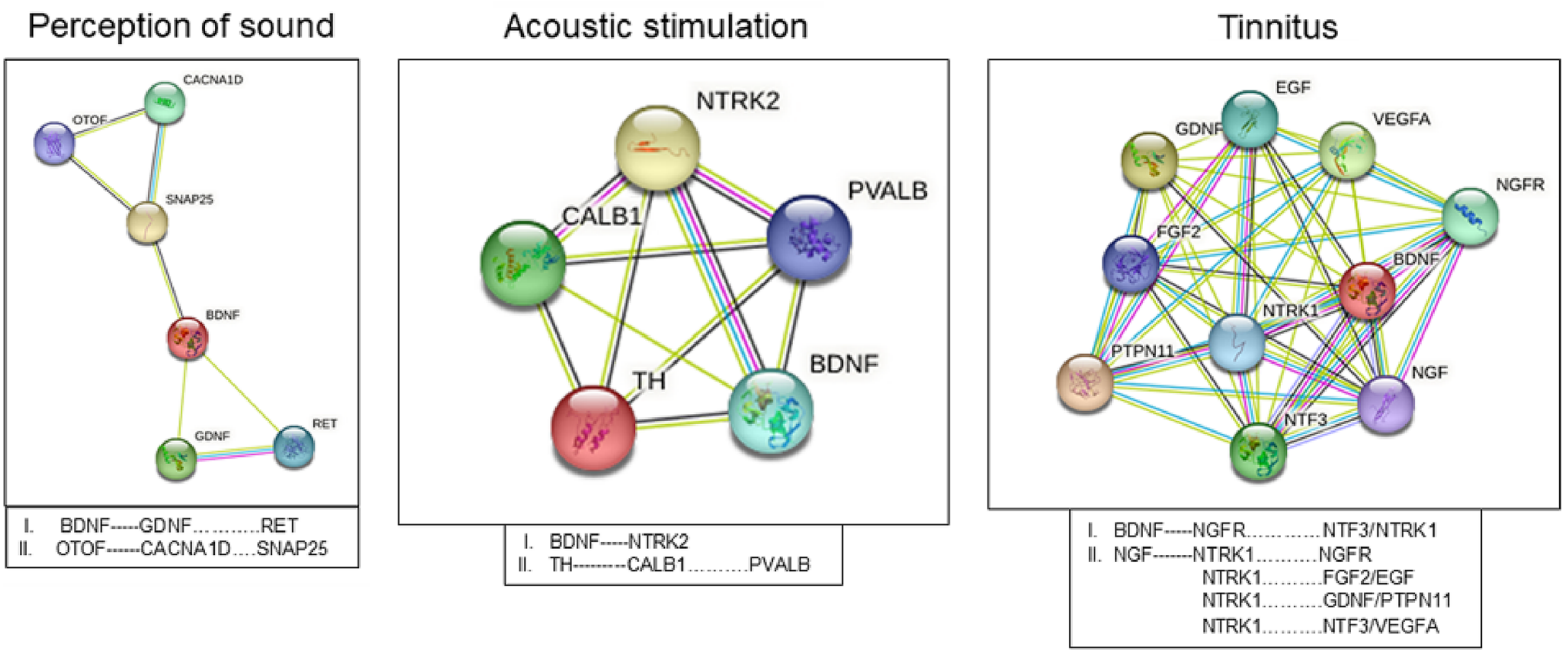
Key proteins and their HSIPs. As a quantitative measure for assessing close interactions, CS values greater than the 90th percentile of the CS corresponding distribution curves were used. Topological parameter (Pos/AcouStim/Tin): nodes 6/5/10; degrees 6-4/8/9-8); edges 14/20/44; average number of neighbors 2.3/4.0/8.8; characteristic path length 1.8/1.0/1.02; network density 0.47/1.0/0.98; network heterogeneity 0.20/0.0/0.05; network centralization 0.20/0.0/0.03

For further characterizing these proteins and to localize them into molecular pathways and biological processes, we searched for proteins that have close interactions with the key proteins. To limit the numbers of proteins, we used as a quantitative measure for assessing close interaction CS values greater than the 90th percentile of the CS distribution curves (CS values: PoS CS > 940, AcouStim CS > 909, Tin CS > 971). The key proteins of the three networks differ significantly in the number and types of proteins with which they form high-score interactions (Fig. 4). In the PoS network, high-score interactions are shown by GDNF with RET and CACNA1D with SNAP25. In the AcouStim network, top2 key protein CALB1 interacts closely with PVALB. In the Tin network, NGFR interacts closely with NTF3, and NTRK1; NTRK1 interacts with six proteins, mostly growth factors (Fig. 4). It is noteworthy that except for BDNF, there is no overlap in the occurrence of these proteins in the PoS, AcouStim, and Tin groups. The networks of these proteins illustrate well the meaning of topological parameters (see legend of Fig. 4). The density of the AcouStim and Tin networks is significantly higher, and the network heterogeneity and centralization are significantly lower, than those of the PoS network.

### GO Terms Associated with Key Proteins

Analysis of GO terms from the complete gene lists showed some overlapping CC- and BP-GO terms in the PoS, AcouStim, and Tin gene lists (Table 2). Assuming that key proteins reflect important cellular structures and biological processes more directly, results of the enrichment analysis should be more informative than of the complete lists, even if the number of proteins is relatively small (PoS + AcouStim *n* = 10, Tin *n* = 10). Irrespective of the same number of proteins in the lists, the numbers of significant charts are clearly higher in the Tin group (*n* = 75) compared to the physiological processes PoS and AcouStim (*n* = 20). The PoS/AcouStim lists contained 6 CC terms and 8 BP terms at a significant level of *p* < 0.01; the Tin list contained 5 CC and 43 BP terms, indicative for the multiple biological processes in SG in Tin compared to PoS/AcouStim (Table 4). The proteins in the PoS and AcouStim processes are related according to the cellular components, similar to the findings of the complete list for axons, dendrites, and the terminal bouton. Regarding the biological processes, the proteins relate to positive regulation of neuron projection development and negative regulation of neuron apoptotic process, and the “brain-derived neurotrophic factor receptor signaling pathway.” The proteins in the Tin process correlate regarding the cellular processes with high significance and, with a high number of proteins, to extracellular processes. Regarding the biological processes, the “nerve growth factor signaling pathway” and developmental processes are dominant.

**Table 4:** Top five Cellular components (CC) and Biological Processes (BP) GO Terms in the spiral ganglion for PoS and AcouStim and Tin processes based on key proteins and their high-score interaction proteins

PoS and AcouStim (10 proteins; 20 charts at *p* < 0.01)	Tin (10 proteins; 75 charts at *p* < 0.01)
*CC (p* = *1.9E-7 to 3.2 E-3–3; fold enrichment 34–135)*	*CC (p* = *4.4E-6 to 1.3E-3; fold enrichment 7–48)*
Axon (BDNF, CALB1, NTRK2, PVALB, RET, TH)	Extracellular region (BDNF, EGF, FGF2, GDNF, NGFR, NGF, NTF3, VEGFA)
Dendrite (BDNF, CALB1, NTRK2, RET, TH)	Extracellular space (BDNF, EGF, FGF2, GDNF, NGF, NTF3, VEGFA)
Terminal bouton (CALB1, NTRK2, TH)	Axon (BDNF, NGF, NTRK1, NTF3)
Synaptic vesicle (BDNF, SNAP25, TH)	Dendrite (BDNF, NGF, NTRK1, NTF3)
Perinuclear region of cytoplasm (BDNF, SNAP25, NTRK2, TH)	Synaptic vesicle (BDNF, NGF, NTF3)
*BP (p* = *1.3E-3 to 2.3E-3; fold enrichment 36–777)*	*BP (p* = *1.5E-8 to 7.3E-7; fold enrichment 28–647)*
Positive regulation of neuron projection development (BDNF, NTRK2, RET)	Nerve growth factor signaling pathway (BDNF, NGF, NTRK1, NTF3)
Transmembrane receptor protein tyrosine kinase signaling pathway (BDNF, NTRK2, RET)	Nerve development (BDNF, NGF, NGFR, NTF3)
Negative regulation of neuron apoptotic process (BDNF, GDNF, NTRK2)	Peripheral nervous system development (BDNF, GDNF, NGF, NTF3)
Brain-derived neurotrophic factor receptor signaling pathway (BDNF, NTRK2)	Negative regulation of neuron apoptotic process (BDNF, GDNF, NGF, NTRK1, NTF3)
Sensory perception of sound (CACNA1D, OTOF, TH)	Nervous system development (BDNF, FGF2, GDNF, NTRK1, NTF3, VEGFA)

### Pathways of the Key Proteins and Their HSIPs

**Fig. 5:**
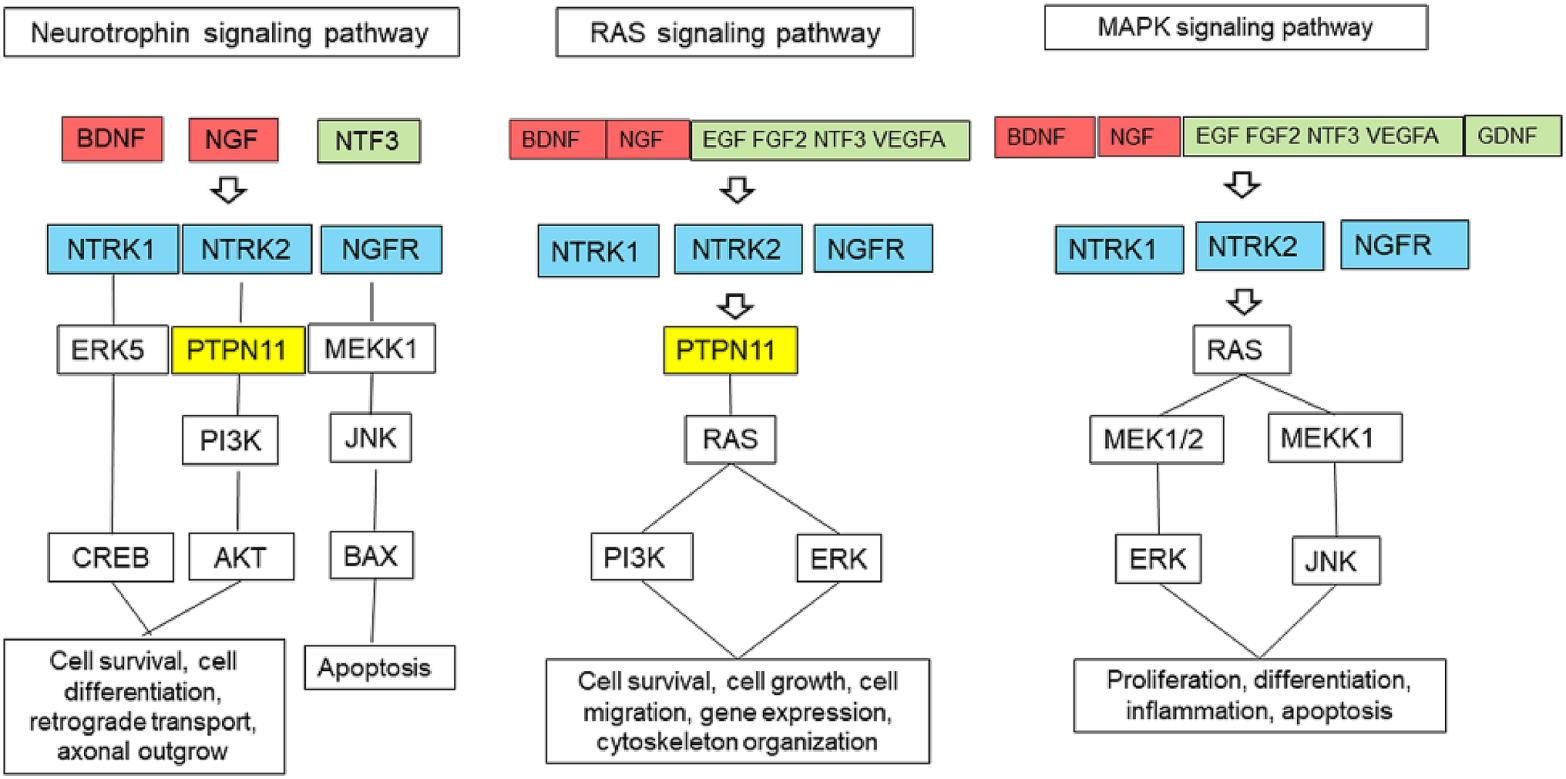
KEGG Pathways associated with key proteins and interaction proteins with a CS > 90th percentile. *ERK5* mitogen-activated protein kinase 12, *PTPN11* tyrosine protein phosphatase non-receptor type 11, also known as SH-PTP2, *PI3K* phosphatidylinositol 3-kinase, *AKT* RAC-gamma serine/threonine protein kinase; *MEKK1* mitogen-activated protein kinase 1, *JNK* mitogen-activated protein kinase 8/9/10 (c-Jun N-terminal kinase), *BAX* Bax inhibitor 1, *RAS* GTPase HRas, *MEK* dual-specificity mitogen-activated protein kinase 1

Key proteins and their closely interacting proteins with a CS > 90th percentile are members of three KEGG pathways: Neurotrophin (map 04722), RAS (map04014), and MAPK (map04010) signaling pathways (https://www.genome.jp/kegg/pathway.html; Fig. 5). Neurotrophins bind to TRK tyrosine kinases (NTRK1, NTRK2, NTRK3) or to the NGFR receptor and act in various cellular functions, such as cell survival, cell proliferation, differentiation, migration, axon growth, gene expression, and apoptosis.

## Discussion

### Neurotrophins and Their Receptors in the SG

To date, 50 neuronal growth factors have been identified, of which BDNF and NGF are of particular importance. The specific roles of BDNF and NGF for some neurons are well known; for example, BDNF promotes the function and survival of cortical neurons, and NGF is known to activate the function of basal forebrain cholinergic neurons and prevents cell death. NGF plays an important role in Alzheimer disease (Tuszynski et al. 2015). The present data suggest that BDNF is the top1 key protein in SG for the three processes PoS, AcouStim, and Tin; NGF has a comparable significance for Tin. Together with neurotrophin-3 (NT3), and neurotrophin-4 (NT4), BDNF and NGF belong to the group of important neurotrophins. Neurotrophins are soluble factors that play an important role in the survival and differentiation of neurons, in axon and dendrite growth, in synaptogenesis, and in synaptic transmission. The function of neurons requires communication between the cell body, axon, dendrites, and synaptic nerve terminals, some of which are spatially distant and require intracellular transport processes to reach the appropriate site (Scott-Solomon and Kuruvilla 2018). Neurotrophins are formed as precursors, the mature form is obtained by proteolytic cleavage. Release can be regulated constitutively and by activation (Fig. 5).

BDNF is involved in all elementary regulatory processes, such as survival and differentiation of neurons, axonal growth, dendrite growth, migration, path finding, transcription, translation, transport, secretion, maturation, differentiation, growth, regeneration or migration, synaptic transmission, and plasticity (Green et al. 2012). An important feature for the multiple influences of BDNF on synaptic transmission and plasticity could be the structure of the BDNF gene, which has eight exons and four different promoters (Reichardt 2006). The expression of each exon can be regulated by its own promoter. Exon IV seems to have a special role; its expression is controlled by calcium-response elements (Singer et al. 2008). Recently, regulation at the microRNA level via BDNF-AS has become increasingly recognized as being important for Tin (Appendix 3; Yuksel et al. 2023). Also has the loss of activity-driven BDNF promotor activation following deafferentation discussed to contribute to central hyperexcitability during tinnitus, through the specific role of BDNF to stabilize inhibitory GABA-ergic circuits (Knipper et al. 2022; Tan et al. 2007).

NGF (Nerve growth factor, also known as NGFB; Beta-NGF) is a member of the NGF-beta family and binds the NTRK1 receptor with high affinity. An important biological function of NGF in the inner ear is to ensure the survival of SGNs, including their dendrites (Dai et al. 2004; Nicoletti et al. 2022).

**Fig. 6:**
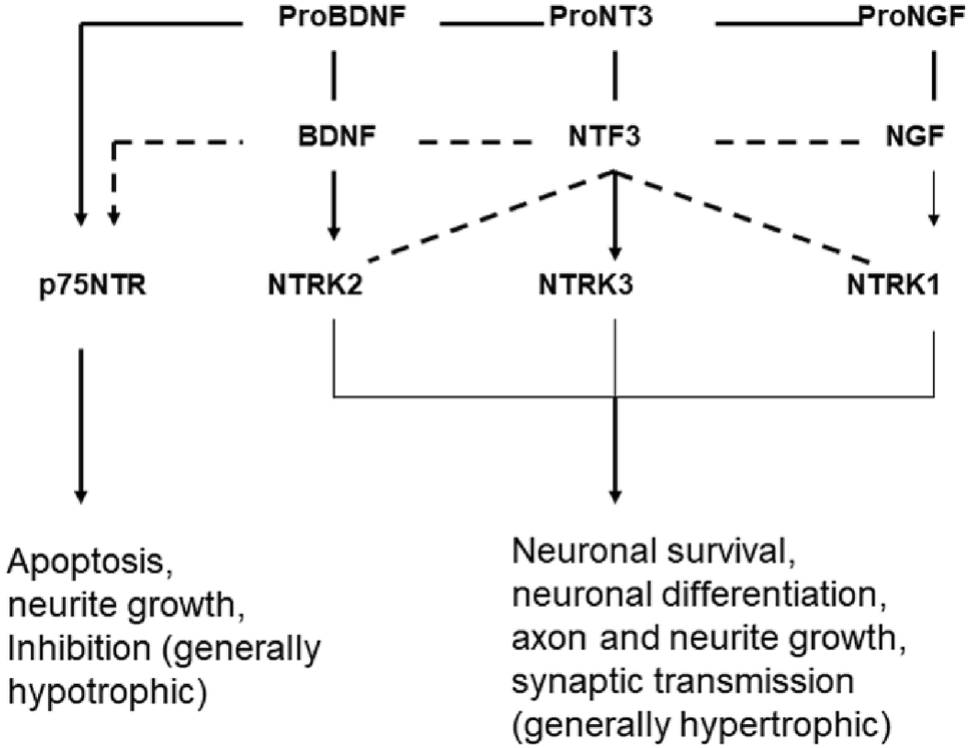
Neurotrophins and their receptors (according to Green et al. 2012)

Neurotrophins exert their function as regulatory proteins for survival and for synaptic transmission by binding and activation of NTRK and NGFR receptors. The TRK family of receptors consists of three members: NTRK1, NTRK2, and NTRK3 (TRKA, TRKB, TRKC). Activation of the receptors by neurotrophins promotes neuronal survival, neuronal differentiation, axon and neurite growth, and synaptic transmission and plasticity. Binding of BDNF alters the structure of the receptor (dimerization, mutual phosphorylation); the receptor thus binds a number of adaptor and signaling proteins and activates, among others, protein kinase B (AKT), mitogen-activated protein kinase (MAPK), and other transcriptional processes via the transcription factor CREB (Nicoletti et al. 2022). The affinity for binding of the different neurotrophins to the kinase receptors differs: BDNF binds and activates mainly NTRK2, NGF-NTRK1, and NT3-NTRK3. NT3 also binds NTRK2 and NTRK1 with weaker affinity (Fig. 6; Scott-Solomon and Kuruvilla 2018; Regua et al. 2019). The expression of neurotrophins and of the receptors influences to a considerable extent the biological effects.

NTRK2 (Neurotrophic Receptor Tyrosine Kinase 2; also known as BDNF/NT3 Growth Factors Receptor, TRKB-Tropomyosin-Related Kinase B) is a cellular component of the postsynaptic membrane and acts together with BDNF in the modulation of synaptic transmission. The BDNF-NTRK2 axis is also involved in the formation of synaptic spines (Zagrebelsky et al. 2020). These are spiny extensions of synapses that allow more precise and spatial signal transmission. They are also part of excitatory synapses in the auditory system and play a major role in synaptic plasticity. The changes in dendritic morphology and their excitability may be associated with the development of tinnitus (Yang et al. 2012). NTRK1 is not a specific component of the synapse and of trans-synaptic signaling. Basically, NTRK1 and NTRK2 activate the same metabolic pathways: PI3K, MAPK, and PLCy (Fig. 6; Regua et al. 2019). Upon dimeric NGF ligand binding, it undergoes homodimerization, autophosphorylation, and activation (Regua et al. 2019). Extracellular ligand NGF binds NTRK1 and NGFR receptors and activates cellular signaling cascades to regulate neuronal proliferation, differentiation, and survival. SGN express NTRK1, NTRK2, and NTRK3 and are protected in vitro by BDNF and NT3 in their function and survival in the face of damaging influences. It is known that after deafferentation of SGN, they die as a result of the loss of hair cells; electrical stimulation promotes survival of SGN after hair cell loss (Hansen et al. 2001).

NGFR (nerve growth factor receptor; also known as P75NTR, p75^NTR^, TNFRSF16 protein tumor necrosis factor receptor superfamily member 16) is a low-affinity receptor for NGF, BDNF, NTF3, and NTF4 (Fig. 5). It is part of the presynaptic membrane and is involved in the modulation of synaptic transmission. In development, NGFR plays an important role in cell differentiation and cell survival and death. While all neurotrophins bind and activate the NGFR receptor only with low affinity, all pro-neurotrophins bind it with high affinity (Green et al. 2012). NGFR is expressed in glial cells as well as neurons; the special glia cell known as the spiral ganglion Schwann cell (SGSCs), which myelinate SGN, represent a source of neurotrophic factors. SGSCs express sortilin, a NGFR co-receptor for pro-neurotrophins (Blondeau et al. 2018; Chen et al. 2005). SGSC cell density appears to be subject to tight regulation of proliferation and cell death upon hair cell injury (Provenzano et al. 2011). In general, NGFR has atrophic effects in the CNS, promoting apoptosis, inhibiting neurite growth, and decreasing synaptic activity.

### Key Proteins of PoS Network

Candidate key proteins of the PoS process are BDNF-GDNF and OTOF-CACNA1D. BDNF and GDNF are extremely important as modulators of synaptic transmission in mature cells of the CNS (Ferrini et al. 2021). Together, BDNF and GDNF are very effective in their actions on survival after ototoxic drugs or noise (Altschuler et al. 1999). GDNF (glial cell line-derived neurotrophic factor) is an important survival and growth factor for SGN (Green et al. 2012). The GDNF receptor is a complex consisting of a member of the GDNF receptor family (GFRa, GDNF family receptor alpha) and the receptor protein tyrosine kinase RET (Fig. 4). RET (proto-oncogene tyrosine protein kinase receptor), a high-score interaction protein of GDNF, is a receptor for neurotrophic factors of the GDNF family. Binding of RET and a member of the GFR family activates fundamental signals for neuronal developmental processes, such as cell proliferation, neuronal navigation, cell migration, cell differentiation, and cell death (Hamada and Lasek 2020). Disruption of c-Ret phosphorylation during development can lead to congenital hearing loss, with degeneration of the SGN in mice (Ohgami et al. 2010).

OTOF (Otoferlin) is a protein that plays a key role in calcium-regulated synaptic transmission from cochlear synapses. Otoferlin simultaneously binds multiple copies of the calcium voltage-gated channel subunit alpha1 D (CACNA1D, also known as Cav1.3 neuronal L-type channel) of the calcium ion channel of neurons and arms of the SNARE (soluble *N*-ethylmaleimide-sensitive factor attachment protein receptor complex) protein complex. Both are required in spatial proximity to allow rapid transmission of the sound signal (Wei et al. 2020). Sound transmission at ribbon synapses between inner hair cells and type1 spiral ganglion neurons are strongly dependent on Ca^2+^-mediated neurotransmitter release mediated by otoferlin and other factors (Takago et al. 2018). CACNA1D is a subunit of calcium channels that regulates the influx of calcium into neural excitable cells and regulates processes, such as neurotransmitter release or gene expression. The alpha1 subunit largely determines the properties of the channel that is important for the translation of sound-induced depolarization into neurotransmitter release. Mutation of the CACNA1D is related to congenital deafness (Baig et al. 2011). Efficient neurotransmitter release requires localization of the calcium channel in the presynaptic terminal near the synaptic vesicle and the SNAP25 release complex (Simms and Zamponi 2014). SNAP25 (synaptosomal-associated protein 25), a high-score interaction protein of CACNA1D, is involved in the molecular regulation of neurotransmitter release, in particular in vesicle docking and membrane fusion (Roux et al. 2006). SNAP25 is a component of the SNARE complex that is closely associated with Otoferlin. Signal transduction in spiral ganglia is under the influence of BDNF and NT3 (Flores-Otero et al. 2007). In the PoS network, SNAP25 and BDNF appear to have a bridging function between the top1 and top2 key proteins (Bolat et al. 2022).

In summary, the proteins BDNF/GDNF/RET and OTOF/CACNA1D/SNAP25 appear to be important in the process of sound perception under control conditions. These proteins represent the biological GO processes of synaptic vesicle priming and synaptic vesicle exocytosis (DAVID). In the PoS process, the protein pair BDNF-GDNF is important for the survival of the cells, and the protein pair OTOF-CACNA1D is important for the fine regulation of synaptic transmission. The network (Fig. 4) suggests that the regulation of survival processes (top1 protein pair) and signaling processes (top2 protein pair) occurs through the interaction of SNAP25 and BDNF.

### Key Proteins of the AcouStim Network

Candidate key proteins of the AcouStim process are BDNF-NTRK2 und TH-CALB1. In comparison to PoS, AcouStim is an activated status of the auditory system. Under these conditions BDNF and its high-affinity receptor NTRK2 become the important modulator of synaptic transmission. The top2 HSIP pair is TH (tyrosine hydroxylase) and CALB1 (Calbindin 1). TH catalyzes the conversion of l-tyrosine to l-dihydroxyphenylalanine (l-Dopa) and that is the rate-limiting step in the biosynthesis of the catecholamines dopamine, noradrenaline, and adrenaline. Dopamine (DA) receptor expression has been demonstrated in the spiral ganglion (Inoue et al. 2006); all five DA receptors were identified on the soma of SGN (Reijntjes and Pyott 2016). The expression of tyrosine hydroxylase was detected in type II afferent neurons of mice (Vyas et al. 2017; Wu et al. 2018). DA is an important neurotransmitter of the lateral efferent dopaminergic system that regulates the excitability of the afferent dendrites of the SGN at the base of the IHC. Wu et al. (2020) demonstrated that DA is also involved in efferent neurons of the lateral olivocochlear region that modulate auditory nerve activity. Inhibition or modulation of BDNF-NTRK2 activity occurs by efferent neurons of the lateral olivocochlear (LOC) system via regulation of TH expression. DA seems to regulate the neurotransmitter release from hair cells and thus the firing rate of SG neurons and to modulate excitatory and inhibitory synaptic transmission pre and postsynaptically (Jacob and Nienborg 2018). A role in protecting the inner ear from excitotoxicity has been widely attributed to dopamine (Lamas et al. 2017).

CALB1 (Calbindin1; also known as CALB; D-28 K) belongs to the group of calcium-binding proteins, such as calmodulin and troponin C. It is involved in the regulation of pre- and postsynaptic cytosolic calcium levels, and it acts as a buffer for calcium in the pre- and postsynaptic cytosol. Studies of CALB1 in the auditory system are not yet available. CALB1 protein regulates DA release and DA transport in the ventral striatum by regulating Ca^++^ influx (Brimblecombe et al. 2019). CALB1 protein is also thought to regulate the activation of glutamate receptors by Ca^++^ influx. Calb1-knockout experiments suggest that CALB1 modifies not only DA release, but also the temporo-spatial coupling of Ca^++^ influx and DA release. CALB1 is involved in the regulation of long-term synaptic potentiation. Raynard et al. (2022) showed that overexpression of CALB1 is protective against increasing levels of Ca^++^ in aging cells. CALB1 interacts closely with PVALB (Parvalbumin), which belongs to the group of Ca^++^-binding proteins that includes calbindin (CALB1) and calretinin (CALB2). Parvalbumin is a small cytosolic protein that binds Ca^++^. Its physiological function is to date insufficiently studied, but it is assumed to be a calcium buffer. Thus, it is able to regulate Ca^++^-dependent metabolic and electrical processes, particularly of gamma-aminobutyric acid (GABA) interneurons (Permyakov and Uversky 2022).

Calcium-binding proteins have been detected in various inhibitory interneurons. The physiological function of these proteins is to regulate Ca^++^ activity, Ca^++^ transport, and the activity of various enzymes. There is evidence that neurons with high concentrations of these Ca^++^ buffers are more resistant to degenerative processes (Idrizbegovic et al. 2006; Schwaller 2020; Permyakov and Uversky 2022). Parvalbumin-positive interneurons belong to the class of GABA-ergic inhibitory neurons of the CNS that have a high axonal conduction velocity. In the cortex, parvalbumin-positive neurons play a major role in plasticity (Rupert and Shea 2022). The expression of PVALB varies depending on the activity of the PVALB-producing neurons. Loss of parvalbumin-positive interneuron activity is involved in the development of tinnitus (Knipper et al. 2020).

In summary, the response of the SG cells to acoustic stimulation is the activation of the BDNF-NTRK2 axis. The activity of the BDNF-NTRK2 system is regulated by the DA-Calcium system. An exaggerated response with cell damage is apparently prevented by regulation via the calcium system. PVALB seems to be of central importance, as it has close interactions with all members of this network (Fig. 4).

### Key Proteins of Tinnitus

#### BDNF, NTRK1, NGF, and NGFR

The key proteins of tinnitus differ from those assigned to PoS and AcouStim not only in the proteins themselves but also by the observation that the HDPs BDNF and NGF show HSIs with NGFR and NTRK1 at approximately the same level of interaction. BDNF and NGF have similar high-degree values (Table 3). In addition, the key proteins interact with a variety of other proteins, of which we have selected here those with the highest CS (Fig. 4). Thus, the key proteins BDNF-NTRK1 and NGF-NGFR interact so closely that it is not possible to distinguish which protein pair has particular dominance. Probably there will be a competition of the receptors for the neurotrophins, thus the concentrations and activities of the receptors or neurotrophins will determine the dominant effect**.**

Both BDNF and NGF play important roles in neurogenesis, differentiation, survival, and synaptic plasticity (Huang and Reichardt 2001). BDNF and NGF can have similar effects on neurons, but at different locations in the cell, depending on the localization of the receptors. However, NGF and BDNF can also have different effects, e.g., NGF can specifically promote survival of cholinergic neurons (Nordvall et al. 2022). This is significant, in that cholinergic input to the hippocampus after noise exposure is important for tinnitus induction (Zhang et al. 2019).

The mechanism of interactions between the four proteins in SG are not well known in detail. It is quite possible that the interaction of BDNF and NTRK1 reflects a connection between BDNF and NGF (1–14), a peptide of the N terminus of NGF (Naletova et al. 2019). Manohar et al. (2016) demonstrated that noise exposure upregulates the mRNA expression of Ntrk1 in the cochlear nucleus as a result of inflammation, neuropathic pain, microglial activation, and the migration of nerve fibers. The interactions of BDNF and NTRK1 might be related to the metabolic effects of NTRK1 on redox status, autophagy, glucose homeostasis, energy, and cholesterol metabolism (Avcı 2021; Colardo et al. 2021; Boecking et al. 2022).

The receptors of BDNF, NTRK2, and NGFR are expressed in SG. The interaction of BDNF and NGFR likely occurs via the action of preBDNF and may lead to apoptosis or inhibition of neurite growth. In primary dissociated SGN, proBDNF has neither toxic nor protective effects. Simultaneous administration of BDNF and proBDNF seems rather to be beneficial for the positive effect of BDNF (Schulze et al. 2022). It is known that SGN degenerate (loss of peripheral and central SGN synapses) without hair cells dying, e.g., in various forms of age-related hearing loss (neural ARHL). Survival of SGN depends strongly on the presence of neurotrophic factors, such as BDNF, NT3, or the glial cell factor GDNF (Shityakov et al. 2021). Several in vitro and in vivo studies show that neurotrophins promote SGN survival and synapse formation (Schulze et al. 2022). It can be assumed that further genetic and environmental factors are responsible for tinnitus (Bao and Ohlemiller 2010).

During embryonic development, NGF and its receptors are significantly expressed in humans. After birth, expression decreases significantly and remains low in adulthood. NGF is involved in the protection of degenerating neurons and in the targeted growth of nerve fibers. Low blood NGF levels seem to be associated with sensorineural hearing loss and tinnitus (Salvinelli et al. 2015). In mouse models, NGF therapy was successful in preserving SGN structure and function (Shityakov et al. 2021). NGF is produced by various cells in the body; it reaches a blood level that may well exert physiological effects.

In the CNS, NGF is present only in the form as proNGF. NGF and the precursor form proNGF appear to be critical for neuronal cell survival and cognitive function. Decreasing NTRK1 expression reduces cell survival; proNGF loses neurotrophic function in the absence of NTRK1, and it promotes apoptosis. Maintaining the balance between NTRK1 and NGFR at axon terminals is critical for the pro-survival or pro-apoptosis effects of proNGF. ProNGF appears to act neurotrophically in the presence of NTRK1, but apoptotically in the absence of Ntrk1 (Kropf and Fahnestock 2021).

NGF is known to promote the survival of basal forebrain cholinergic nuclei neurons in vitro and in vivo. NGF is produced in the cortex and hippocampus and is retrogradely transported to cholinergic neurons of the basal forebrain. These neurons play a major role in cognition and attention and in Alzheimer’s disease (Shityakov et al. 2021). It acts through two cell surface receptors, NTRK1 and NGFR; this involves the formation of a trimeric complex and the activation of signaling pathways that promote cell survival. Binding of NGF to the receptors can occur at axon terminals. Transport into the cell body involves retrograde transport (Canu et al. 2017). The problem with NGF therapy is that NGF is a large and polar protein and cannot cross the blood–brain barrier when administered peripherally. NGF has severe negative effects on some neurons, e.g., loss of pain perception (Tuszynski et al. 2015). Because NGF is detectable in physiologically effective amounts in the blood and NTRK1 receptors are expressed in the spiral ganglion, therapeutic trials have been undertaken for sensorineural hearing loss and tinnitus (Zhou et al. 2009; Salvinelli et al. 2015). Disturbances in the secretion of these neurotrophins lead to irreversible degeneration of the SGN. In contrast to mature NGF, the interaction of the precursor form (proNGF) and sortilin promotes neuronal apoptosis (Tauris et al. 2011; Vaegter et al. 2011; Carlo 2013).

#### High-Score Interaction Proteins

The four key proteins (BDNF, NTRK1, NGF, NGFR) show high-score interactions to NTF3, to several growth factors (FGF2, EGF, GDNF, NTF3, VEGFA), and to regulatory proteins (PTPN11; Fig. 4). *Neurotrophin 3* (NTF3, NT3) belongs to the neurotrophin family, with NTRK3 as the high-affinity receptor (Fig. 5). NTF3 binds and activates NTRK1 and NTRK2 with weaker affinity (Green et al. 2012). In the developmental phase, NTF3 is a critical factor for the survival of auditory neurons and for the formation of ribbon synapses on inner hair cells (Wan et al. 2014). It is known for its regulation of the formation of IHC-SGN synapses in the neonatal period (Cassinotti et al. 2022). NTF3 also plays a role in the recovery of synaptic connections after noise trauma (Wan et al. 2014). A close interaction of FGF2 and NTF3 was demonstrated in a hippocampal neuron culture (Igelhorst et al. 2015). Under the influence of NTF3, NTRK3 over- or under-expression affected synaptic density; NTF3 is considered to be a key protein for the formation of synapses (Han et al. 2016).

*Fibroblast growth factor 2* (FGF2, also FGFB) belongs to a large family of growth factors (23 members) with paracrine and endocrine effects on angiogenesis, metabolism, proliferation, survival, migration, and differentiation. Together with NTF3, it is involved in the proliferation of SGNs and in the formation of axons, nerve terminals, and synaptic vesicles (Hossain et al. 2008). In mouse SG, FGF2 and GDNF were shown to play an important role in the long-term survival of neurons after separation of the SG from hair cells. A combination of FGF2 and GDNF could be used as a therapeutic measure for the secondary degeneration of SGN (Wei et al. 2007). In the SG, FGF2 plays a major role in the survival and regeneration of neurons in cooperation with GDNF (glial cell line-derived neurotrophic factor). FGF2 has a particular importance for the regeneration of neurites (Wei et al. 2007). After the detection of neural stem cells in SG, there were attempts to induce these SGN stem cells to differentiate into neural and glial cells. This required the interaction of valproic acid and FGF2 (Moon et al. 2018). Using a guinea pig model, FGF2 was shown to protect against glutamate excitotoxicity and acoustic trauma (Zhai et al. 2004).

The *protein tyrosine phosphatase non-receptor type 11* (PTPN11) gene encodes protein tyrosine phosphatase (also SHP2), a member of the protein tyrosine phosphatase (PTP) family. It is involved in signal transduction for cell growth, differentiation, metabolic control, regulation of transcription, and cell migration; the protein interacts with phospholipids and phosphoproteins (Abbasi et al. 2018; Gao et al. 2021; Wu et al. 2023). The non-receptor protein tyrosine phophatase PTPN11 has two catalytic domains and is ubiquitously expressed (Wu et al. 2023). Patients with a pathogenic PTPN11 mutation exhibit congenital sensorineural hearing loss (Gao et al. 2021). PTPN11 has been shown to be a critical factor in learning, memory, and plasticity (Zhang et al. 2017). Thus, PTPN11 may contribute significantly to synaptic homeostasis. PTPN11 (Protein Tyrosine Phosphatase Non-Receptor Type 11) appears to be an important switch point in the pathway toward cell survival and axonal outgrowth (Fig. 5).

*Vascular endothelial growth factor A* (VEGFA) is a growth factor active in angiogenesis, vasculogenesis, and endothelial cell growth. VEGF is active in angiogenesis and especially in endothelial cell growth. In addition to promoting angiogenesis, VEGFA has growth-promoting effects on glial cells and neurons. In various tissues, there is a very close interaction between FGF2 and VEGFA to form new blood vessels (Idrisova et al. 2022). The interaction between FGF2 and VEGFA might also be related to the biological role of perlecan (Hayes et al. 2022; Nguyen et al. 2021). Perlecan protein (heparan sulfate proteoglycan 2, HSPG2) has five different domains and is an integral component of basement membranes; it serves as an attachment substrate for cells and plays essential roles in vascularization (Niemeyer et al. 2020). The effects of FGF2 and VEGFA in the nervous system were recently summarized in a comprehensive review (Idrisova et al. 2022). Neuro- and angiotrophic growth factors play a key role in the regeneration of neurons and their processes. FGF2 and VEGFA have been implicated in improving cognitive performance and neurogenesis in the hippocampus and the frontal cortex in the rat after exercise (Chen et al. 2022). In an auditory PPI network of Phoenix sphere cells, FGF2 and VEGFA were identified as hub genes (Sølvsten et al. 2018; Rousset et al. 2020).

*Epidermal growth factor* (EGF) belongs to the EGF superfamily and is an important mitogenic factor important for growth, proliferation, and differentiation of numerous cell types. EGF is expressed in SG neuron cells and peripheral processes during development and adulthood (Hartman et al. 2010). It could play a role in the differentiation of human bone marrow-derived mesenchymal stem cells to auditory hair cells (Mahmoudian-Sani et al. 2017).

In summary, BDNF and NGF interact in tinnitus with NTRK1/NGFR in a competitive, modulatory manner influencing development, degeneration, and regeneration of neuronal and non-neuronal tissue. In addition, a variety of growth factors interacting closely with the key proteins is involved in the modulation of the signaling system. In tinnitus, these changes suggest processes of degeneration and regeneration of neural and non-neural tissues and a reorganization of synaptic transmission processes in the SG. For a potential therapy, it is important to reduce or normalize the massive reorganization, growth, and regeneration processes in the SG.

## Conclusion

The current study has limitations: (1) The list of genes was compiled on the basis of keywords that can be assigned to the symptom “tinnitus” (independently of whether subjective or objective) or to the processes “perception of sound” and “acoustic stimulation.” Overlaps or overestimates of specific genes cannot be excluded. (2) The precise effect of genes and proteins in a given disease is often tissue- and cell-type dependent (Greene et al. 2015). The databases used here contain global metabolic and molecular functions and do not distinguish among species, tissue, cell type, or pathogenesis (Wong et al. 2018). The exact effects of proteins often depend on their tissue context, but direct tests for tissue-specific protein functions and interactions in many normal human tissues and cell types remain impossible. (3) As key proteins, we have selected only the top two proteins based on the degree and their high-score interacting proteins. It is not always possible to clearly decide which protein is top1 or top2. (4) The composition of the gene list is crucial for finding key proteins and HSIP. Our approach assumes that the genes of the respective list play a role in the process under investigation in terms of up- or down-regulation or in influencing the activity of other proteins. What role these proteins play in tinnitus requires experimental clarification.

Comparison of the key proteins of PoS, AcouStim, and Tin shows that analysis of the top1 and top2 HDPs and their HSIPs can be valuable for identifying candidate key proteins and basic molecular processes for clinical symptoms and diseases. In PoS (normal conditions), the top1 protein pair (BDNF-GDNF) is directed toward maintaining cell structure and function. The top2 protein pair (OTOF-CACNA1D) is directed to synaptic fine regulation. In AcouStim (activated status), the top1 protein pair (BDNF-NTRK2) is directed to the activation of the neuronal reactions; the BDNF-NTRK2 protein pair plays an important role in synaptic transmission under physiological activated conditions. The top2 protein pair (TH-CALB1) is directed to the counter-reaction via DA and Ca^++^ and may protect the system from excess workload and thus cell damage. The BDNF-NTRK2 interactions run in the PoS process in the background, without special activation or change. In Tin, both top1 and top2 protein pairs are targeted to growth and regeneration processes. The focus is on cell death and cell regeneration and thus constant readjustment of synaptic processes. This regulation takes place at cell and tissue level and refers to neural and non-neural cells. In the PoS and AcouStim networks, the BDNF receptor is the dominant signaling pathway; in the Tin network, the NGF-signaling pathway attains similar importance. BDNF and NGF, in their interaction with NTRK1/NGFR, are involved in cell growth, survival, and regeneration, with the consequence of the changes in synaptic transmission connected with tinnitus.

The results of the current study provide new insights into the molecular biology of tinnitus and could be important for the detection of proteins with opposing or modulating effects. From a clinical perspective, further improvements in diagnosis and therapy of tinnitus are needed. Current research fields are focused on elucidation of molecular-biological mechanisms, biomarkers of tinnitus distress, psychological distress (Henry et al. 2014; Basso et al. 2022a, b; Boecking et al. 2022), and psychotherapeutic treatment methods (Mazurek et al. 2022). For this, a unified data collection (Schlee et al. 2021) and advances in big-data analysis are of increasing importance, with the goal of paving the way toward a personalized diagnosis and therapy of tinnitus.

## Data Availability

The datasets generated and analyzed during the current study are publicly available in the databases indicated.

## Appendix 1: Genes Identified by the Keywords “Perception of Sound” AND “Synaptic Transmission” AND “Spiral Ganglion” (*n* = 36)

**Table:**

Gene	Score	Name
BDNF	18.86	Brain-Derived Neurotrophic Factor
SNAP25	12.42	Synaptosome-Associated Protein 25
MAP1B	11.74	Microtubule-Associated Protein 1B
GDNF	10.8	Glial Cell-Derived Neurotrophic Factor
P2RX2	10.03	Purinergic Receptor P2X 2
CHAT	9.67	Choline *O*-Acetyltransferase
GRM7	7.04	Glutamate Metabotropic Receptor 7
TH	6.43	Tyrosine Hydroxylase
CDH23	6.1	Cadherin-Related 23
KCNQ4	6.07	Potassium Voltage-Gated Channel Subfamily Q Member 4
GRM8	5.84	Glutamate Metabotropic Receptor 8
MT-CO3*	5.75	Mitochondrially Encoded Cytochrome C Oxidase III
DES*	5.16	Desmin
CACNA1D	5.11	Calcium Voltage-Gated Channel Subunit Alpha1 D
SLC17A8	4.75	Solute Carrier Family 17 Member 8
ASIC2	4.65	Acid Sensing Ion Channel Subunit 2
RET	4.57	Ret Proto-Oncogene
GJA1	4.16	Gap Junction Protein Alpha 1
MBP	4.09	Myelin Basic Protein
OTOF	3.53	Otoferlin
WHRN	3.09	Whirlin
MYO7A	2.81	Myosin VIIA
CA2	2.58	Carbonic Anhydrase 2
KCNJ10	2.55	Potassium Inwardly Rectifying Channel Subfamily J Member 10
CPA6*	2.53	Carboxypeptidase A6
NRTN	2.4	Neurturin
SLC26A5	2.23	Solute Carrier Family 26 Member 5
SETD2*	2.08	SET Domain Containing 2, Histone Lysine Methyltransferase
NOG	2.03	Noggin
TBC1D24	1.79	TBC1 Domain Family Member 24
PTPN11	1.76	Protein Tyrosine Phosphatase Non-Receptor Type 11
LMNB1*	1.74	Lamin B1
MPDZ*	1.71	Multiple PDZ Domain Crumbs Cell Polarity Complex Component
MAP2K1	1.47	Mitogen-Activated Protein Kinase Kinase 1
BTD*	1.37	Biotinidase
MAFB*	1.09	MAF BZIP Transcription Factor B

*Not in the PPI network

## Appendix 2: Genes Identified by the Keywords “Acoustic Stimulation” AND “Synaptic Transmission” AND “Spiral Ganglion” (*n* = 34)

**Table:**

Gene	Score	Name
BDNF-AS RNA		RNA Gene
BDNF	20.82	Brain-Derived Neurotrophic Factor
MALAT1*	10.5	Metastasis-Associated Lung Adenocarcinoma Transcript 1
NTRK2	9.2	Neurotrophic Receptor Tyrosine Kinase 2
PVALB	9.18	Parvalbumin
P2RX2*	9	Purinergic Receptor P2X 2
SYP	8.15	Synaptophysin
NR3C1	7.72	Nuclear Receptor Subfamily 3 Group C Member 1
SMAD5-AS1*	6.8	SMAD5 Antisense RNA 1
CERNA3*	6.46	Competing Endogenous LncRNA 3 For MiR-645
GFAP	6.43	Glial Fibrillary Acidic Protein
CALB1	6.36	Calbindin 1
TH	6.12	Tyrosine Hydroxylase
CREB1	5.95	CAMP-Responsive Element Binding Protein 1
CNTF	5.8	Ciliary Neurotrophic Factor
SOD2-OT1*	5.68	SOD2 Overlapping Transcript 1
CDH23	5.54	Cadherin-Related 23
NOS1	5.23	Nitric Oxide Synthase 1
CALB2	5.1	Calbindin 2
KCNQ4	5.04	Potassium Voltage-Gated Channel Subfamily Q Member 4
NRG1	4.74	Neuregulin 1
RET	4.53	Ret Proto-Oncogene
NTS	4.52	Neurotensin
MAPK10	4.29	Mitogen-Activated Protein Kinase 10
EGF	4.11	Epidermal Growth Factor
CACNA1D	4.08	Calcium Voltage-Gated Channel Subunit Alpha1 D
OPRM1	4.06	Opioid Receptor Mu 1
MAP2	3.93	Microtubule-Associated Protein 2
TMX2-CTNND1*	3.46	TMX2-CTNND1 Read-through (NMD Candidate)
OTOF	3.4	Otoferlin
NOS2	2.95	Nitric Oxide Synthase 2
CYP19A1	1.71	Cytochrome P450 Family 19 Subfamily A Member 1
JUN	1.28	Jun Proto-Oncogene, AP-1 Transcription Factor Subunit
SLC26A5	1.04	Solute Carrier Family 26 Member 5

*Not in the PPI network

## Appendix 3: Genes Identified by the Keywords “Tinnitus” AND “Synaptic Transmission” AND “Spiral Ganglion” (*n* = 61)

**Table:**

Gene	Score	Name
BDNF-AS*	30.86	BDNF Antisense RNA
BDNF	23.4	Brain-Derived Neurotrophic Factor
CDH23	14.91	Cadherin-Related 23
NTF3	14.62	Neurotrophin 3
MALAT1*	14.35	Metastasis-Associated Lung Adenocarcinoma Transcript 1
GDNF	13.86	Glial Cell-Derived Neurotrophic Factor
SNAP25	12.51	Synaptosome-Associated Protein 25
MAP1B	11.46	Microtubule-Associated Protein 1B
P2RX2	11.34	Purinergic Receptor P2X 2
MYO7A	10.85	Myosin VIIA
CAMK2G	10.46	Calcium/Calmodulin-Dependent Protein Kinase II Gamma
RET	10.44	Ret Proto-Oncogene
KCNQ4	10.37	Potassium Voltage-Gated Channel Subfamily Q Member 4
SYP	8.63	Synaptophysin
NR3C1	7.31	Nuclear Receptor Subfamily 3 Group C Member 1
CACNA1D	7.28	Calcium Voltage-Gated Channel Subunit Alpha1 D
GFAP	6.95	Glial Fibrillary Acidic Protein
NGF	6.8	Nerve Growth Factor
TH	6.14	Tyrosine Hydroxylase
GPHN	6.05	Gephyrin
NGFR	6.04	Nerve Growth Factor Receptor
NTRK3	5.86	Neurotrophic Receptor Tyrosine Kinase 3
GRM7	5.77	Glutamate Metabotropic Receptor 7
CREB1	5.32	CAMP-Responsive Element Binding Protein 1
HIF1A	5.01	Hypoxia-Inducible Factor 1 Subunit Alpha
CALB2	4.44	Calbindin 2
FGF2	4.22	Fibroblast Growth Factor 2
MAP2	4.2	Microtubule-Associated Protein 2
SLC17A8	4.08	Solute Carrier Family 17 Member 8
EGF	4.06	Epidermal Growth Factor
NOS2	4.03	Nitric Oxide Synthase 2
MBP	3.94	Myelin Basic Protein
NRG1	3.79	Neuregulin 1
NTS	3.71	Neurotensin
GJA1	3.63	Gap Junction Protein Alpha 1
PCAT1*	3.63	Prostate Cancer-Associated Transcript 1
ANXA5	3.57	Annexin A5
SLC26A5	3.55	Solute Carrier Family 26 Member 5
WHRN	3.11	Whirlin
VEGFA	3.07	Vascular Endothelial Growth Factor A
CACNA1G	2.87	Calcium Voltage-Gated Channel Subunit Alpha1 G
NTRK1	2.71	Neurotrophic Receptor Tyrosine Kinase 1
KCNJ10	2.62	Potassium Inwardly Rectifying Channel Subfamily J Member 10
OTOF	2.61	Otoferlin
MAP2K1	2.51	Mitogen-Activated Protein Kinase 1
S100B	2.35	S100 Calcium-Binding Protein B
PTPN11	2.33	Protein Tyrosine Phosphatase Non-Receptor Type 11
CA2	2.16	Carbonic Anhydrase 2
GFRA1	2.14	GDNF Family Receptor Alpha 1
NRTN	2.13	Neurturin
MIR210*	2.12	MicroRNA 210
NUDT6	1.94	Nudix Hydrolase 6
VIM	1.88	Vimentin
SETD2	1.66	SET Domain Containing 2, Histone Lysine Methyltransferase
TBC1D24	1.51	TBC1 Domain Family Member 24
PANX1	1.29	Pannexin 1
ID1	1.19	Inhibitor Of DNA Binding 1
BTD*	1.09	Biotinidase
JUN	1.06	Jun Proto-Oncogene, AP-1 Transcription Factor Subunit
USF1	0.94	Upstream Transcription Factor 1
MAFB	0.81	MAF BZIP Transcription Factor B

*Not in the PPI network

## References

[CR1] Abbasi M, Gupta V, Chitranshi N et al (2018) Regulation of brain-derived neurotrophic factor and growth factor signaling pathways by tyrosine phosphatase Shp2 in the retina: a brief review. Front Cell Neurosci 12:85. 10.3389/fncel.2018.00085 29636665 10.3389/fncel.2018.00085 PMC5880906

[CR2] Altschuler RA, Cho Y, Ylikoski J et al (1999) Rescue and regrowth of sensory nerves following deafferentation by neurotrophic factors. Ann N Y Acad Sci 884:305–311. 10.1111/j.1749-6632.1999.tb08650.x 10842602 10.1111/j.1749-6632.1999.tb08650.x

[CR3] Ashtiani M, Salehzadeh-Yazdi A, Razaghi-Moghadam Z et al (2018) A systematic survey of centrality measures for protein-protein interaction networks. BMC Syst Biol 12:80. 10.1186/s12918-018-0598-2 30064421 10.1186/s12918-018-0598-2 PMC6069823

[CR4] Avcı D (2021) Increased serum lipid levels in patients with subjective tinnitus. Iran J Otorhinolaryngol 33:31–36. 10.22038/ijorl.2020.43663.2442 33654688 10.22038/ijorl.2020.43663.2442 PMC7897436

[CR5] Baguley D, McFerran D, Hall D (2013) Tinnitus. Lancet Lond Engl 382:1600–1607. 10.1016/S0140-6736(13)60142-7 10.1016/S0140-6736(13)60142-7 23827090

[CR6] Baig SM, Koschak A, Lieb A et al (2011) Loss of Ca(v)1.3 (CACNA1D) function in a human channelopathy with bradycardia and congenital deafness. Nat Neurosci 14:77–84. 10.1038/nn.2694 21131953 10.1038/nn.2694

[CR7] Bao J, Ohlemiller KK (2010) Age-related loss of spiral ganglion neurons. Hear Res 264:93–97. 10.1016/j.heares.2009.10.009 19854255 10.1016/j.heares.2009.10.009 PMC2868093

[CR8] Basso L, Boecking B, Neff P et al (2022a) Hair-cortisol and hair-BDNF as biomarkers of tinnitus loudness and distress in chronic tinnitus. Sci Rep 12:1934. 10.1038/s41598-022-04811-0 35121746 10.1038/s41598-022-04811-0 PMC8817043

[CR9] Basso L, Boecking B, Neff P et al (2022b) Psychological treatment effects unrelated to hair-cortisol and hair-BDNF levels in chronic tinnitus. Front Psychiatry 13:764368. 10.3389/fpsyt.2022.764368 35250657 10.3389/fpsyt.2022.764368 PMC8895295

[CR10] Blondeau N, Béraud-Dufour S, Lebrun P et al (2018) Sortilin in glucose homeostasis: from accessory protein to key player? Front Pharmacol 9:1561. 10.3389/fphar.2018.01561 30697159 10.3389/fphar.2018.01561 PMC6340931

[CR11] Boecking B, Klasing S, Walter M et al (2022) Vascular-metabolic risk factors and psychological stress in patients with chronic tinnitus. Nutrients 14:2256. 10.3390/nu14112256 35684056 10.3390/nu14112256 PMC9183085

[CR12] Bolat H, Ünsel-Bolat G, Özgül S et al (2022) Investigation of possible associations of the BDNF, SNAP-25 and SYN III genes with the neurocognitive measures: BDNF and SNAP-25 genes might be involved in attention domain, SYN III gene in executive function. Nord J Psychiatry 76:610–615. 10.1080/08039488.2022.2027518 35077325 10.1080/08039488.2022.2027518

[CR13] Brimblecombe KR, Vietti-Michelina S, Platt NJ et al (2019) Calbindin-D28K limits dopamine release in ventral but not dorsal striatum by regulating Ca2+ availability and dopamine transporter function. ACS Chem Neurosci 10:3419–3426. 10.1021/acschemneuro.9b00325 31361457 10.1021/acschemneuro.9b00325 PMC6706870

[CR14] Canu N, Amadoro G, Triaca V et al (2017) The intersection of NGF/TrkA signaling and amyloid precursor protein processing in Alzheimer’s disease neuropathology. Int J Mol Sci 18:1319. 10.3390/ijms18061319 28632177 10.3390/ijms18061319 PMC5486140

[CR15] Carlo A-S (2013) Sortilin, a novel APOE receptor implicated in Alzheimer disease. Prion 7:378–382. 10.4161/pri.26746 24121631 10.4161/pri.26746 PMC4134342

[CR16] Cassinotti LR, Ji L, Borges BC et al (2022) Cochlear neurotrophin-3 overexpression at mid-life prevents age-related inner hair cell synaptopathy and slows age-related hearing loss. Aging Cell 21:e13708. 10.1111/acel.13708 36088647 10.1111/acel.13708 PMC9577954

[CR17] Chen Z-Y, Ieraci A, Teng H et al (2005) Sortilin controls intracellular sorting of brain-derived neurotrophic factor to the regulated secretory pathway. J Neurosci Off J Soc Neurosci 25:6156–6166. 10.1523/JNEUROSCI.1017-05.2005 10.1523/JNEUROSCI.1017-05.2005 PMC1201519 15987945

[CR18] Chen J, Liu Z, Chang J (2022) Genetic mechanism study of auditory phoenix spheres and transcription factors prediction for direct reprogramming by bioinformatics. Int J Mol Sci 23:10287. 10.3390/ijms231810287 36142199 10.3390/ijms231810287 PMC9499413

[CR19] Colardo M, Martella N, Pensabene D et al (2021) Neurotrophins as key regulators of cell metabolism: implications for cholesterol homeostasis. Int J Mol Sci 22:5692. 10.3390/ijms22115692 34073639 10.3390/ijms22115692 PMC8198482

[CR20] Dai CF, Steyger PS, Wang ZM et al (2004) Expression of Trk A receptors in the mammalian inner ear. Hear Res 187:1–11. 10.1016/s0378-5955(03)00277-6 14698082 10.1016/s0378-5955(03)00277-6 PMC3964775

[CR21] Eggermont JJ, Roberts LE (2015) Tinnitus: animal models and findings in humans. Cell Tissue Res 361:311–336. 10.1007/s00441-014-1992-8 25266340 10.1007/s00441-014-1992-8 PMC4487353

[CR22] Ferrini F, Salio C, Boggio EM, Merighi A (2021) Interplay of BDNF and GDNF in the mature spinal somatosensory system and its potential therapeutic relevance. Curr Neuropharmacol 19:1225–1245. 10.2174/1570159X18666201116143422 33200712 10.2174/1570159X18666201116143422 PMC8719296

[CR23] Flores-Otero J, Xue HZ, Davis RL (2007) Reciprocal regulation of presynaptic and postsynaptic proteins in bipolar spiral ganglion neurons by neurotrophins. J Neurosci Off J Soc Neurosci 27:14023–14034. 10.1523/JNEUROSCI.3219-07.2007 10.1523/JNEUROSCI.3219-07.2007 PMC6673512 18094241

[CR24] Gao X, Huang S-S, Qiu S-W et al (2021) Congenital sensorineural hearing loss as the initial presentation of PTPN11-associated Noonan syndrome with multiple lentigines or Noonan syndrome: clinical features and underlying mechanisms. J Med Genet 58:465–474. 10.1136/jmedgenet-2020-106892 32737134 10.1136/jmedgenet-2020-106892

[CR25] Green SH, Bailey E, Wang Q, Davis RL (2012) The Trk A, B, C’s of neurotrophins in the cochlea. Anat Rec 295:1877–1895. 10.1002/ar.22587 10.1002/ar.22587 23044862

[CR26] Greene CS, Krishnan A, Wong AK et al (2015) Understanding multicellular function and disease with human tissue-specific networks. Nat Genet 47:569–576. 10.1038/ng.3259 25915600 10.1038/ng.3259 PMC4828725

[CR27] Hamada K, Lasek AW (2020) Receptor tyrosine kinases as therapeutic targets for alcohol use disorder. Neurother J Am Soc Exp Neurother 17:4–16. 10.1007/s13311-019-00795-4 10.1007/s13311-019-00795-4 PMC7007457 31617071

[CR28] Han KA, Woo D, Kim S et al (2016) Neurotrophin-3 regulates synapse development by modulating TrkC-PTPσ synaptic adhesion and intracellular signaling pathways. J Neurosci Off J Soc Neurosci 36:4816–4831. 10.1523/JNEUROSCI.4024-15.2016 10.1523/JNEUROSCI.4024-15.2016 PMC6601719 27122038

[CR29] Hansen MR, Zha XM, Bok J, Green SH (2001) Multiple distinct signal pathways, including an autocrine neurotrophic mechanism, contribute to the survival-promoting effect of depolarization on spiral ganglion neurons in vitro. J Neurosci Off J Soc Neurosci 21:2256–2267 10.1523/JNEUROSCI.21-07-02256.2001 PMC6762385 11264301

[CR30] Hartman BH, Nelson BR, Reh TA, Bermingham-McDonogh O (2010) Delta/notch-like EGF-related receptor (DNER) is expressed in hair cells and neurons in the developing and adult mouse inner ear. J Assoc Res Otolaryngol 11:187–201. 10.1007/s10162-009-0203-x 20058045 10.1007/s10162-009-0203-x PMC2862923

[CR31] Hayes AJ, Farrugia BL, Biose IJ et al (2022) Perlecan, A multi-functional, cell-instructive, matrix-stabilizing proteoglycan with roles in tissue development has relevance to connective tissue repair and regeneration. Front Cell Dev Biol 10:856261. 10.3389/fcell.2022.856261 35433700 10.3389/fcell.2022.856261 PMC9010944

[CR32] Henry JA, Roberts LE, Caspary DM et al (2014) Underlying mechanisms of tinnitus: review and clinical implications. J Am Acad Audiol 25:5–22. 10.3766/jaaa.25.1.2. (**quiz 126**) 24622858 10.3766/jaaa.25.1.2 PMC5063499

[CR33] Hickman TT, Hashimoto K, Liberman LD, Liberman MC (2021) Cochlear synaptic degeneration and regeneration after noise: effects of age and neuronal subgroup. Front Cell Neurosci 15:684706. 10.3389/fncel.2021.684706 34434091 10.3389/fncel.2021.684706 PMC8380781

[CR34] Hossain WA, D’Sa C, Morest DK (2008) Interactive roles of fibroblast growth factor 2 and neurotrophin 3 in the sequence of migration, process outgrowth, and axonal differentiation of mouse cochlear ganglion cells. J Neurosci Res 86:2376–2391. 10.1002/jnr.21685 18438927 10.1002/jnr.21685

[CR35] Huang EJ, Reichardt LF (2001) Neurotrophins: roles in neuronal development and function. Annu Rev Neurosci 24:677–736. 10.1146/annurev.neuro.24.1.677 11520916 10.1146/annurev.neuro.24.1.677 PMC2758233

[CR36] Idrisova KF, Zeinalova AK, Masgutova GA et al (2022) Application of neurotrophic and proangiogenic factors as therapy after peripheral nervous system injury. Neural Regen Res 17:1240–1247. 10.4103/1673-5374.327329 34782557 10.4103/1673-5374.327329 PMC8643040

[CR37] Idrizbegovic E, Salman H, Niu X, Canlon B (2006) Presbyacusis and calcium-binding protein immunoreactivity in the cochlear nucleus of BALB/c mice. Hear Res 216–217:198–206. 10.1016/j.heares.2006.01.009 16874908 10.1016/j.heares.2006.01.009

[CR38] Igelhorst BA, Niederkinkhaus V, Karus C et al (2015) Regulation of neuronal excitability by release of proteins from glial cells. Philos Trans R Soc Lond B 370:20140194. 10.1098/rstb.2014.0194 26009773 10.1098/rstb.2014.0194 PMC4455763

[CR39] Inoue T, Matsubara A, Maruya S et al (2006) Localization of dopamine receptor subtypes in the rat spiral ganglion. Neurosci Lett 399:226–229. 10.1016/j.neulet.2006.01.063 16490310 10.1016/j.neulet.2006.01.063

[CR40] Isler B, von Burg N, Kleinjung T et al (2022) Lower glutamate and GABA levels in auditory cortex of tinnitus patients: a 2D-JPRESS MR spectroscopy study. Sci Rep 12:4068. 10.1038/s41598-022-07835-8 35260698 10.1038/s41598-022-07835-8 PMC8904839

[CR41] Jacob SN, Nienborg H (2018) Monoaminergic neuromodulation of sensory processing. Front Neural Circ 12:51. 10.3389/fncir.2018.00051 10.3389/fncir.2018.00051 PMC6048220 30042662

[CR42] Knipper M, van Dijk P, Schulze H et al (2020) The neural bases of tinnitus: lessons from deafness and cochlear implants. J Neurosci Off J Soc Neurosci 40:7190–7202. 10.1523/JNEUROSCI.1314-19.2020 10.1523/JNEUROSCI.1314-19.2020 PMC7534911 32938634

[CR43] Knipper M, Singer W, Schwabe K, Hagberg GE, Li Hegner Y, Rüttiger L, Braun C, Land R (2022) Disturbed balance of inhibitory signaling links hearing loss and cognition. Front Neural Circ 15:785603. 10.3389/fncir.2021.785603 10.3389/fncir.2021.785603 PMC8770933 35069123

[CR44] Kropf E, Fahnestock M (2021) Effects of reactive oxygen and nitrogen species on TrkA expression and signalling: implications for proNGF in aging and Alzheimer’s disease. Cells 10:1983. 10.3390/cells10081983 34440751 10.3390/cells10081983 PMC8392605

[CR45] Lamas V, Juiz JM, Merchán MA (2017) Ablation of the auditory cortex results in changes in the expression of neurotransmission-related mRNAs in the cochlea. Hear Res 346:71–80. 10.1016/j.heares.2017.02.011 28216123 10.1016/j.heares.2017.02.011

[CR46] Lefebvre PP, Staecker H, Van de Water T et al (2002) Pharmacologic treatment of inner ear: from basic science to the patient. Acta Otorhinolaryngol Belg 56:45–49 11894630

[CR47] Mahmoudian-Sani M-R, Hashemzadeh-Chaleshtori M et al (2017) In vitro differentiation of human bone marrow mesenchymal stem cells to hair cells using growth factors. Int Tinnitus J 21:179–184. 10.5935/0946-5448.20170030 29336137 10.5935/0946-5448.20170030

[CR48] Manohar S, Dahar K, Adler HJ et al (2016) Noise-induced hearing loss: neuropathic pain via Ntrk1 signaling. Mol Cell Neurosci 75:101–112. 10.1016/j.mcn.2016.07.005 27473923 10.1016/j.mcn.2016.07.005 PMC5002254

[CR49] Mazurek B, Rose M, Schulze H, Dobel C (2022) Systems medicine approach for tinnitus with comorbid disorders. Nutrients 14:4320. 10.3390/nu14204320 36297004 10.3390/nu14204320 PMC9611054

[CR50] Moon B-S, Lu W, Park HJ (2018) Valproic acid promotes the neuronal differentiation of spiral ganglion neural stem cells with robust axonal growth. Biochem Biophys Res Commun 503:2728–2735. 10.1016/j.bbrc.2018.08.032 30119886 10.1016/j.bbrc.2018.08.032

[CR51] Naletova I, Satriano C, Pietropaolo A et al (2019) The Copper(II)-assisted connection between NGF and BDNF by means of nerve growth factor-mimicking short peptides. Cells 8:E301. 10.3390/cells8040301 10.3390/cells8040301 PMC6523629 30939824

[CR52] Nayagam BA, Muniak MA, Ryugo DK (2011) The spiral ganglion: connecting the peripheral and central auditory systems. Hear Res 278:2–20. 10.1016/j.heares.2011.04.003 21530629 10.1016/j.heares.2011.04.003 PMC3152679

[CR53] Nguyen B, Bix G, Yao Y (2021) Basal lamina changes in neurodegenerative disorders. Mol Neurodegener 16:81. 10.1186/s13024-021-00502-y 34876200 10.1186/s13024-021-00502-y PMC8650282

[CR54] Nicoletti VG, Pajer K, Calcagno D et al (2022) The role of metals in the neuroregenerative action of BDNF, GDNF, NGF and other neurotrophic factors. Biomolecules 12:1015. 10.3390/biom12081015 35892326 10.3390/biom12081015 PMC9330237

[CR55] Niemeyer C, Matosin N, Kaul D et al (2020) The role of cathepsins in memory functions and the pathophysiology of psychiatric disorders. Front Psychiatry 11:718. 10.3389/fpsyt.2020.00718 32793006 10.3389/fpsyt.2020.00718 PMC7393989

[CR56] Nordvall G, Forsell P, Sandin J (2022) Neurotrophin-targeted therapeutics: a gateway to cognition and more? Drug Discov Today 27:103318. 10.1016/j.drudis.2022.07.003 35850433 10.1016/j.drudis.2022.07.003

[CR57] Ohgami N, Ida-Eto M, Shimotake T et al (2010) c-Ret-mediated hearing loss in mice with Hirschsprung disease. Proc Natl Acad Sci USA 107:13051–13056. 10.1073/pnas.1004520107 20616061 10.1073/pnas.1004520107 PMC2919946

[CR58] Pavlinkova G (2020) Molecular aspects of the development and function of auditory neurons. Int J Mol Sci 22:E131. 10.3390/ijms22010131 10.3390/ijms22010131 PMC7796308 33374462

[CR59] Permyakov EA, Uversky VN (2022) What is parvalbumin for? Biomolecules 12:656. 10.3390/biom12050656 35625584 10.3390/biom12050656 PMC9138604

[CR60] Provenzano MJ, Minner SA, Zander K et al (2011) p75(NTR) expression and nuclear localization of p75(NTR) intracellular domain in spiral ganglion Schwann cells following deafness correlate with cell proliferation. Mol Cell Neurosci 47:306–315. 10.1016/j.mcn.2011.05.010 21658451 10.1016/j.mcn.2011.05.010 PMC3137691

[CR61] Raynard C, Tessier N, Huna A et al (2022) Expression of the calcium-binding protein CALB1 is induced and controls intracellular Ca2+ levels in senescent cells. Int J Mol Sci 23:9376. 10.3390/ijms23169376 36012633 10.3390/ijms23169376 PMC9409414

[CR62] Regua AT, Doheny D, Arrigo A, Lo H-W (2019) Trk receptor tyrosine kinases in metastasis and cancer therapy. Discov Med 28:195–203 31928627

[CR63] Reichardt LF (2006) Neurotrophin-regulated signalling pathways. Philos Trans R Soc Lond B 361:1545–1564. 10.1098/rstb.2006.1894 16939974 10.1098/rstb.2006.1894 PMC1664664

[CR64] Reijntjes DOJ, Pyott SJ (2016) The afferent signaling complex: regulation of type I spiral ganglion neuron responses in the auditory periphery. Hear Res 336:1–16. 10.1016/j.heares.2016.03.011 27018296 10.1016/j.heares.2016.03.011

[CR65] Resnik J, Polley DB (2021) Cochlear neural degeneration disrupts hearing in background noise by increasing auditory cortex internal noise. Neuron 109:984-996.e4. 10.1016/j.neuron.2021.01.015 33561398 10.1016/j.neuron.2021.01.015 PMC7979519

[CR66] RoussetKokje FBCV, Sipione R et al (2020) Intrinsically self-renewing neuroprogenitors from the A/J mouse spiral ganglion as virtually unlimited source of mature auditory neurons. Front Cell Neurosci 14:395. 10.3389/fncel.2020.599152 33362466 10.3389/fncel.2020.599152 PMC7761749

[CR67] Roux I, Safieddine S, Nouvian R et al (2006) Otoferlin, defective in a human deafness form, is essential for exocytosis at the auditory ribbon synapse. Cell 127:277–289. 10.1016/j.cell.2006.08.040 17055430 10.1016/j.cell.2006.08.040

[CR68] Rupert DD, Shea SD (2022) Parvalbumin-positive interneurons regulate cortical sensory plasticity in adulthood and development through shared mechanisms. Front Neural Circ 16:886629. 10.3389/fncir.2022.886629 10.3389/fncir.2022.886629 PMC9120417 35601529

[CR69] Salvinelli F, Frari V, Rocco ML et al (2015) Enhanced presence of NGF and mast cells number in nasal cavity after autologous stimulation: relation with sensorineural hearing deficit. Eur Rev Med Pharmacol Sci 19:381–391 25720707

[CR70] Schlee W, Schoisswohl S, Staudinger S et al (2021) Towards a unification of treatments and interventions for tinnitus patients: the EU research and innovation action UNITI. Prog Brain Res 260:441–451. 10.1016/bs.pbr.2020.12.005 33637231 10.1016/bs.pbr.2020.12.005

[CR71] Schulze J, Staecker H, Wedekind D et al (2022) Expression pattern of brain-derived neurotrophic factor and its associated receptors: implications for exogenous neurotrophin application. Hear Res 413:108098. 10.1016/j.heares.2020.108098 33143996 10.1016/j.heares.2020.108098

[CR72] Schwaller B (2020) Cytosolic Ca^2+^ buffers are inherently Ca^2+^ signal modulators. Cold Spring Harb Perspect Biol 12:a035543. 10.1101/cshperspect.a035543 31308146 10.1101/cshperspect.a035543 PMC6942125

[CR73] Scott-Solomon E, Kuruvilla R (2018) Mechanisms of neurotrophin trafficking via Trk receptors. Mol Cell Neurosci 91:25–33. 10.1016/j.mcn.2018.03.013 29596897 10.1016/j.mcn.2018.03.013 PMC6128733

[CR74] Shannon P, Markiel A, Ozier O et al (2003) Cytoscape: a software environment for integrated models of biomolecular interaction networks. Genome Res 13:2498–2504. 10.1101/gr.1239303 14597658 10.1101/gr.1239303 PMC403769

[CR75] Sherman BT, Huang DW, Tan Q et al (2007) DAVID Knowledgebase: a gene-centered database integrating heterogeneous gene annotation resources to facilitate high-throughput gene functional analysis. BMC Bioinform 8:426. 10.1186/1471-2105-8-426 10.1186/1471-2105-8-426 PMC2186358 17980028

[CR76] Shityakov S, Hayashi K, Störk S et al (2021) The conspicuous link between ear, brain and heart-could neurotrophin-treatment of age-related hearing loss help prevent Alzheimer’s disease and associated amyloid cardiomyopathy? Biomolecules 11:900. 10.3390/biom11060900 34204299 10.3390/biom11060900 PMC8235707

[CR77] Simms BA, Zamponi GW (2014) Neuronal voltage-gated calcium channels: structure, function, and dysfunction. Neuron 82:24–45. 10.1016/j.neuron.2014.03.016 24698266 10.1016/j.neuron.2014.03.016

[CR78] Singer W, Panford-Walsh R, Watermann D et al (2008) Salicylate alters the expression of calcium response transcription factor 1 in the cochlea: implications for brain-derived neurotrophic factor transcriptional regulation. Mol Pharmacol 73:1085–1091. 10.1124/mol.107.041814 18198284 10.1124/mol.107.041814

[CR79] Sølvsten CAE, de Paoli F, Christensen JH, Nielsen AL (2018) Voluntary physical exercise induces expression and epigenetic remodeling of VegfA in the rat hippocampus. Mol Neurobiol 55:567–582. 10.1007/s12035-016-0344-y 27975171 10.1007/s12035-016-0344-y

[CR80] Stelzer G, Plaschkes I, Oz-Levi D et al (2016) VarElect: the phenotype-based variation prioritizer of the GeneCards suite. BMC Genomics 17(Suppl 2):444. 10.1186/s12864-016-2722-2 27357693 10.1186/s12864-016-2722-2 PMC4928145

[CR81] Sun S, Babola T, Pregernig G et al (2018) Hair cell mechanotransduction regulates spontaneous activity and spiral ganglion subtype specification in the auditory system. Cell 174:1247–1263. 10.1016/j.cell.2018.07.008 30078710 10.1016/j.cell.2018.07.008 PMC6429032

[CR82] Szklarczyk D, Gable AL, Lyon D et al (2019) STRING v11: protein-protein association networks with increased coverage, supporting functional discovery in genome-wide experimental datasets. Nucleic Acids Res 47:D607–D613. 10.1093/nar/gky1131 30476243 10.1093/nar/gky1131 PMC6323986

[CR83] Takago H, Oshima-Takago T, Moser T (2018) Disruption of otoferlin alters the mode of exocytosis at the mouse inner hair cell ribbon synapse. Front Mol Neurosci 11:492. 10.3389/fnmol.2018.00492 30687007 10.3389/fnmol.2018.00492 PMC6338019

[CR84] Tan J, Rüttiger L, Panford-Walsh R et al (2007) Tinnitus behavior and hearing function correlate with the reciprocal expression patterns of BDNF and Arg3.1/arc in auditory neurons following acoustic trauma. Neuroscience 145:715–726. 10.1016/j.neuroscience.2006.11.067 17275194 10.1016/j.neuroscience.2006.11.067

[CR85] Tauris J, Gustafsen C, Christensen EI et al (2011) Proneurotrophin-3 may induce Sortilin-dependent death in inner ear neurons. Eur J Neurosci 33:622–631. 10.1111/j.1460-9568.2010.07556.x 21261755 10.1111/j.1460-9568.2010.07556.x PMC3078644

[CR86] Turrigiano G (2012) Homeostatic synaptic plasticity: local and global mechanisms for stabilizing neuronal function. Cold Spring Harb Perspect Biol 4:a005736. 10.1101/cshperspect.a005736 22086977 10.1101/cshperspect.a005736 PMC3249629

[CR87] Tuszynski MH, Yang JH, Barba D et al (2015) Nerve growth factor gene therapy: activation of neuronal responses in Alzheimer disease. JAMA Neurol 72:1139–1147. 10.1001/jamaneurol.2015.1807 26302439 10.1001/jamaneurol.2015.1807 PMC4944824

[CR88] Tziridis K, Friedrich J, Brüeggemann P et al (2022) Estimation of tinnitus-related socioeconomic costs in Germany. Int J Environ Res Public Health 19:10455. 10.3390/ijerph191610455 36012089 10.3390/ijerph191610455 PMC9407899

[CR89] Vaegter CB, Jansen P, Fjorback AW et al (2011) Sortilin associates with Trk receptors to enhance anterograde transport and neurotrophin signaling. Nat Neurosci 14:54–61. 10.1038/nn.2689 21102451 10.1038/nn.2689 PMC3808973

[CR90] Vyas P, Wu JS, Zimmerman A et al (2017) Tyrosine hydroxylase expression in type II cochlear afferents in mice. J Assoc Res Otolaryngol 18:139–151. 10.1007/s10162-016-0591-7 27696081 10.1007/s10162-016-0591-7 PMC5243262

[CR91] Wan G, Gómez-Casati ME, Gigliello AR et al (2014) Neurotrophin-3 regulates ribbon synapse density in the cochlea and induces synapse regeneration after acoustic trauma. eLife 3:e03564. 10.7554/eLife.03564 25329343 10.7554/eLife.03564 PMC4227045

[CR92] Wei D, Jin Z, Järlebark L et al (2007) Survival, synaptogenesis, and regeneration of adult mouse spiral ganglion neurons in vitro. Dev Neurobiol 67:108–122. 10.1002/dneu.20336 17443776 10.1002/dneu.20336

[CR93] Wei M, Wang W, Liu Y et al (2020) Protection of cochlear ribbon synapses and prevention of hidden hearing loss. Neural Plast 2020:8815990. 10.1155/2020/8815990 33204247 10.1155/2020/8815990 PMC7652619

[CR94] Wong AK, Krishnan A, Troyanskaya OG (2018) GIANT 2.0: genome-scale integrated analysis of gene networks in tissues. Nucleic Acids Res 46:W65–W70. 10.1093/nar/gky408 29800226 10.1093/nar/gky408 PMC6030827

[CR95] Wu C, Wu X, Yi B et al (2018) Changes in GABA and glutamate receptors on auditory cortical excitatory neurons in a rat model of salicylate-induced tinnitus. Am J Transl Res 10:3941–3955 30662641 PMC6325520

[CR96] Wu PZ, Liberman LD, Bennett K et al (2019) Primary neural degeneration in the human cochlea: evidence for hidden hearing loss in the aging ear. Neuroscience 407:8–20. 10.1016/j.neuroscience.2018.07.053 30099118 10.1016/j.neuroscience.2018.07.053 PMC6369025

[CR97] Wu JS, Yi E, Manca M et al (2020) Sound exposure dynamically induces dopamine synthesis in cholinergic LOC efferents for feedback to auditory nerve fibers. eLife 9:e52419. 10.7554/eLife.52419 31975688 10.7554/eLife.52419 PMC7043886

[CR98] Wu X, Huang M, Huang W et al (2023) Preliminary investigation of the diagnosis and gene function of deep learning PTPN11 gene mutation syndrome deafness. Front Genet 14:1113095. 10.3389/fgene.2023.1113095 36760995 10.3389/fgene.2023.1113095 PMC9907458

[CR99] Yang S, Su W, Bao S (2012) Long-term, but not transient, threshold shifts alter the morphology and increase the excitability of cortical pyramidal neurons. J Neurophysiol 108:1567–1574. 10.1152/jn.00371.2012 22723674 10.1152/jn.00371.2012 PMC3544952

[CR100] Yuksel B, Dogan M, Boyacioglu O et al (2023) Association between chronic tinnitus and brain-derived neurotrophic factor antisense RNA polymorphisms linked to the Val66Met polymorphism in BDNF. Gene 875:147507. 10.1016/j.gene.2023.147507 37230202 10.1016/j.gene.2023.147507

[CR101] Zagrebelsky M, Tacke C, Korte M (2020) BDNF signaling during the lifetime of dendritic spines. Cell Tissue Res 382:185–199. 10.1007/s00441-020-03226-5 32537724 10.1007/s00441-020-03226-5 PMC7529616

[CR102] Zeng F-G (2020) Tinnitus and hyperacusis: central noise, gain and variance. Curr Opin Physiol 18:123–129. 10.1016/j.cophys.2020.10.009 33299958 10.1016/j.cophys.2020.10.009 PMC7720792

[CR103] Zhai S-Q, Wang D-J, Wang J-L et al (2004) Basic fibroblast growth factor protects auditory neurons and hair cells from glutamate neurotoxicity and noise exposure. Acta Otolaryngol 124:124–129. 10.1080/00016480310015939 15072413 10.1080/00016480310015939

[CR104] Zhang Z, Lei A, Xu L et al (2017) Similarity in gene-regulatory networks suggests that cancer cells share characteristics of embryonic neural cells. J Biol Chem 292:12842–12859. 10.1074/jbc.M117.785865 28634230 10.1074/jbc.M117.785865 PMC5546026

[CR105] Zhang R, Guo H, Yang X et al (2019) Pathway-based network analyses and candidate genes associated with Kashin-Beck disease. Medicine 98:e15498. 10.1097/MD.0000000000015498 31045836 10.1097/MD.0000000000015498 PMC6504273

[CR106] Zhang L, Chen S, Sun Y (2021) Mechanism and prevention of spiral ganglion neuron degeneration in the cochlea. Front Cell Neurosci 15:814891. 10.3389/fncel.2021.814891 35069120 10.3389/fncel.2021.814891 PMC8766678

[CR107] Zhou F, Wu P, Wang L et al (2009) The NGF point-injection for treatment of the sound-perceiving nerve deafness and tinnitus in 68 cases. J Tradit Chin Med Chung Tsa Chih Ying Wen Pan 29:39–42. 10.1016/s0254-6272(09)60029-7 10.1016/s0254-6272(09)60029-7 19514187

